# Characterizing the Conformational Dynamics of the Ribose Transporter B Protein in *Escherichia coli*: Enhanced Sampling via Multiple Force Fields

**DOI:** 10.1021/acs.jctc.5c02068

**Published:** 2026-02-25

**Authors:** Nikolai Juraschko, Florencia Klein Rocha, Syma Khalid

**Affiliations:** † Department of Biochemistry, 6396 University of Oxford, Oxford OX1 3QU, U.K.; ‡ Rosalind Franklin Institute, Harwell Science & Innovation Campus, Didcot OX11 0QX, U.K.

## Abstract

We present a molecular dynamics simulation study of the *E. coli* ribose transporter protein B (RbsB), a conformationally labile protein found in the periplasm of the bacterium. The ribose transporter exhibits characteristics of both traditional type I and type II import systems. In our study, we observed the full conformational transition of the periplasmic binding protein RbsB for the first time. Our study revealed that in most scenarios (all but one) the conformational changes preceded the departure of ribose from the binding site, a process likely influenced by specific interactions at the binding interface. Indeed, our analyses of ribose binding revealed that specific salt bridges played a crucial role in stabilizing the closed conformation of RbsB. Our simulations also provided further evidence for a putative structural water molecule, which had also been observed from X-ray data. Crucially, our simulations were run with three different force fields: CHARMM36­(m), AMBER ff19SB, and CHARMM36­(m) with SIRAH coarse-grained water. This strategy enabled us to observe all of the conformational states that had been identified in structural studies. Thus, we argue that the subtle biases of individual force fields can be utilized to enhance conformational sampling.

## Graphical abstract

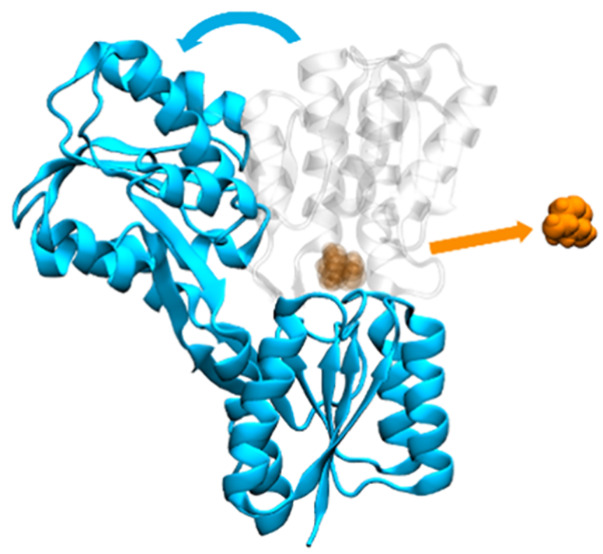

## Introduction

Substrate-binding proteins (SBPs) constitute a class of proteins that vary in size and sequence, yet their three-dimensional structural fold is well conserved. Their general conformation consists of two structurally conserved domains connected by a hinge region.^1^ In the absence of a ligand, the protein adopts an open conformation, exposing the binding site. Upon ligand binding, the protein undergoes a conformational change, trapping the ligand within the cleft between the two domains.^2,3^ This structural rearrangement enables the binding protein to interact with membrane proteins.

In Gram-negative bacteria, SBPs are located in the periplasm^4^ and are subclassified as periplasmic binding proteins (PBPs). These proteins play a role in protein folding and protection against stress in the periplasm.^5^ PBPs are characterized by a conserved quaternary structure known as the bilobal structural fold.^6,7^ Their ability to bind multiple ligands, such as sugars, amino acids, peptides, ions, and vitamins^3^ allows them to mediate a variety of essential processes such as transport, chemotaxis, and quorum sensing.^1,8,9^ Most PBPs facilitate the transport of solute molecules into the cytoplasm via ABC transporters.^1,10^ In Gram-negative bacteria such as *Escherichia coli*, the ribose transporter (RbsABC2) plays a crucial role in the high-affinity uptake of ribose, which is essential for nucleic acid synthesis.^11^

SBPs are classified into distinct structural and functional groups; those associated with type I importers undergo more pronounced conformational changes upon substrate binding compared to those of type II.^6^ The ribose transporter exhibits characteristics of both type I and type II import systems.^12^ In particular, ribose transporter protein B (RbsB) is categorized as belonging to type I.^2,13^

The mechanism of ribose binding into RbsB involves several steps, beginning with the diffusion of ribose into the periplasm through outer membrane porins.^14^ RbsB captures free ribose and subsequently delivers it to the inner membrane complex formed by RbsAC, which then translocates the ribose across the membrane into the cytoplasm via primary active transport.^15,16^

RbsB is an α/β protein composed of two nearly identical globular domains. The N-terminal domain is formed by two separate peptide segments that include four α-helices and four β-strands. The C-terminal domain contains five α-helices and four β-strands. These two domains are linked by three β-strands, creating a flexible three-hinge region (residues 98–108, 229–239, and 260–270), which allows them to move relative to each other upon ligand binding at the domain interface.^2,17^ This categorizes them as cluster B, according to the classification by Berntsson et al.,^6^ as it is an SBP in which the C- and N-termini are not located within the same domain, with three hinges separating the two domains^6^ (see Figure 1).

**Figure 1:**
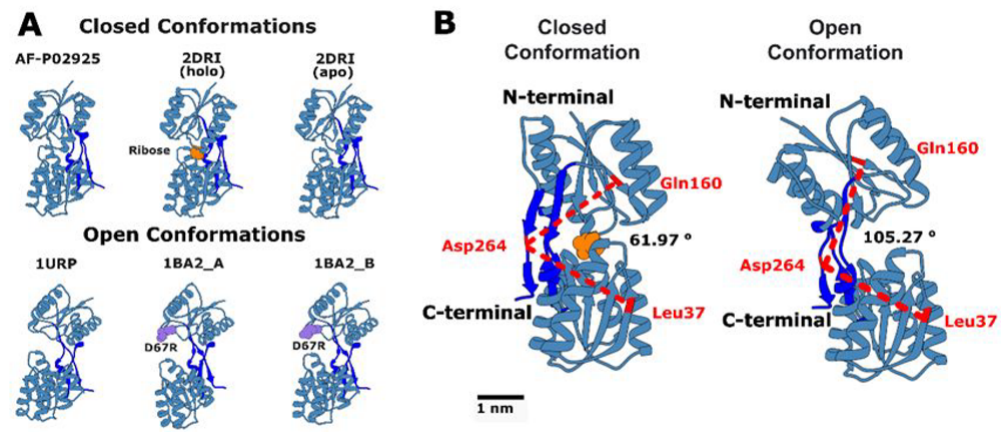
(A) Initial conformations used in the RbsB simulations. The closed states shown are apo AF-P02925, holo 2DRI, and apo 2DRI. The open states are apo 1URP and the D67R mutant 1BA2, conformations A and B. The names correspond to the PDB codes. Key features are colored: ribose (orange), the D67R mutation site (purple), and the three hinge regions (dark blue; residues 98 to 108, 229 to 239, and 260 to 270). (B) Definition of the interdomain opening angle, measured between Cα atoms of Leu37 and Gln160, located in the β-sheets of the two separate main domains, and Asp264, positioned in the hinge region connecting the two domains. A closed, ribose-bound conformation (left) and an open apo conformation (right) are shown as examples.

Understanding the motion around the multistrand hinge region and the pathway of conformational transition from the bound state to the ligand-free state is of interest, given such changes are crucial for the proper functioning of bacterial periplasmic receptors involved in chemotaxis and transport. Over the past two decades, numerous studies have investigated the different motions of the protein, yet full understanding of the interconversion process and whether there is a consistent opening pattern remains elusive.^1,2,12,17–20^ To address this, in the present study the closed holo (ribose-bound) and apo wild-type (wt) form of RbsB (PDB code 2DRI)^19^ are examined, as well as the open apo wt form (PDB code 1URP),^2^ the open apo AlphaFold structure (AF-P02925), and two open apo forms with a mutation at D67R (PDB code 1BA2,^2^ referred to as mutants A and B, respectively; see Figure 1). The D67R mutation is known to impact the transport function of the protein but not its role in chemotaxis.^18^

Molecular dynamics (MD) simulations enable exploration of protein structure-dynamics relationships by capturing conformational fluctuations, binding events, and large-scale motions that are often inaccessible to static experimental methods.^21,22^ MD simulations, while providing information about dynamics at high resolution, are restricted to short time scales, and therefore, the conformational landscapes are often incompletely sampled. Here we have attempted to improve the sampling by as well as carrying out multiple simulations (the *de riguere* approach), also using three different force fields (FFs). The rationale being that the subtle variations in FFs may enable more efficient exploration of the protein conformational landscape. Importantly, this strategy is not intended as a substitute for formal enhanced sampling techniques, such as umbrella sampling, metadynamics, or replica exchange, but rather to reduce FF-specific bias and assess the robustness of mechanistic features across independent physical models. The underlying rationale is that subtle variations in FFs parametrizations may stabilize different regions of conformational space and broaden qualitative exploration of relevant substates.

This approach is conceptually aligned with recent methodological analyses demonstrating that ensembles obtained from many short and independent MD replicates can, in some cases, restore thermodynamic sampling in systems with rugged free-energy landscapes, complementing more formal enhanced sampling strategies.^23^ Such multiple replicate strategies can exploit the diversity of initial conditions and model parametrizations to sample a wider range of conformational substates, without the need for explicit biasing variables. To test validity of this approach, we assess and compare three popular FF–water model combinations for simulations of soluble proteins: the AMBER ff19SB FF with the optimal point charge (OPC) water model,^24,25^ the CHARMM36­(m) FF^26^ with the TIP3P^27^ water model, and the CHARMM36­(m) FF with the TIP3P and SIRAH WatFour (WT4) bulk water models^28–30^ (hereafter denoted as SIRAH hybrid).

## Methods

The AMBER FFs have been shown to correctly fold and preserve the structural integrity of diverse protein systems, including protein–ligand complexes.^31–34^ The most recent FF for proteins in this family, ff19SB FF, introduced new backbone dihedral parameters with amino-acid-specific CMAP functions derived from quantum mechanical calculations in solution.^25,35^ While ff19SB itself is not strictly coupled to a particular water model, the OPC water model has been recommended^24,25^ due to its superior reproduction of key bulk water properties relative to other rigid models like TIP3P.^36^

CHARMM36­(m) is a refinement of the CHARMM36 FF, with modifications to the backbone CMAP potential and an improved description of specific salt bridge interactions to better capture the behavior of both structured and intrinsically disordered proteins.^37^ In this work, CHARMM36­(m) is used with the TIP3P water model, a computationally efficient three-site model that, despite known shortcomings, remains widely used.^36,38^

The SIRAH hybrid framework was implemented by describing the protein with CHARMM36­(m) and water solvation shells near the protein with TIP3P water, while bulk water was represented at coarse-grained (CG) resolution with WT4 molecules.^28–30^ The CG WT4 water model consists of four beads arranged in a tetrahedral conformation. This hybrid approach maintains essential solute–solvent interactions, while enabling access to longer time scales and larger system sizes without sacrificing critical molecular detail.

Several different systems are reported in this study; therefore, to facilitate the navigation of the paper, they were summarized in Table 1 and their detailed setup was described below. Unless otherwise stated, all MD simulations were performed using the GROMACS (version 2023.0) simulation suite or higher^39^ and three different FFs: The AMBER ff19SB FF^25^ in combination with the recommended OPC water model,^24,25^ the CHARMM36­(m) FF with the TIP3P water model,^40^ and a SIRAH hybrid FF which uses the CHARMM36­(m) FF with TIP3P water for all-atom parts of the simulation and the SIRAH WT4 bulk water (see Table 1).^28–30^

**Table 1:** Summary of Simulation Parameters, Including the Simulation Engine, Force Field, Water Model, Temperature, Starting Structure, and Number of Replicas (1 μs each)

				number of 1 μs simulations per system					
Simulation Engine	Force Field	Water Model	*T* (K)	2DRI (holo)	2DRI (apo)	AF-P02925	1URP	1BA2_B	1BA2_A
GROMACS	AMBER ff19SB	OPC	303	7	5	5	5	5	5
	CHARMM36(m)	TIP3P	303	7	5	5	5	5	5
	SIRAH hybrid (CHARMM36 for all-atom parts)	TIP3P + WT4	303	7	5	5	5	5	5

### System Setup

We performed five independent replicas for each of the three FFs considered and each of the six protein structures, respectively, except for 2DRI with ribose, for which we conducted seven replicas each. Each replica was run for 1 μs, giving a total simulation time of 102 μs. Five of the six protein structures—2DRI (with and without ribose) (1.6 Å resolution),^19^ 1BA2 (chains A and B) (2.1 Å), and 1URP (2.3 Å)^2^—were obtained from their corresponding X-ray crystal structures with the same RCSB PDB IDs. The structure AF-P02925 was obtained from the AlphaFold protein structure database.^41,42^ AlphaFold-predicted structures are widely used as reliable starting conformations for MD simulations, especially when high-resolution experimental structures are unavailable. Consequently, the AlphaFold model served as an alternative starting conformation to assess its suitability for MD simulations and capacity to reproduce relevant conformational dynamics. The signal peptides were removed from each protein based on the sequence information from the UniProt database.^43^ The CHARMM and AMBER simulations were prepared using the CHARMM-GUI.^44^ The SIRAH hybrid simulations were set up with GROMACS utilities—the systems contained the protein structure with a TIP3P water shell. Any water molecules further than 1 nm from the protein structure were replaced with WT4 CG water beads before energy minimization.^28–30^ Each simulation box contained a single protein structure and had initial dimensions of 9 nm × 9 nm × 9 nm. Each simulation system was solvated in 150 mM NaCl after addition of neutralizing counterions.

### Molecular Dynamics

The LINCS algorithm^45,46^ was used for bonds to reset coupled constraints after an unconstrained update. The van der Waals interactions for the CHARMM and SIRAH hybrid FF simulations were smoothed at distances beyond 1.0 nm to a cutoff at 1.2 nm. The van der Waals interactions were considered up to a plain cutoff of 1.0 nm for the AMBER FF simulations, with a plain cutoff being less expensive to calculate than the force-switch of the previous simulations. Long-range electrostatics were treated using the particle mesh Ewald (PME) method^47^ with a cutoff distance of 1.2 nm for the CHARMM and SIRAH hybrid FFs. The smooth PME method was also used for AMBER systems, but with a shorter cutoff distance of 1.0 nm. All simulations were performed at 303 K (central to the range of temperatures where *E. coli* survives)^48^ with a coupling constant of τ = 1 ps maintained by Nosé–Hoover dynamics.^49^ All systems were energy minimized in 5000 steps using the steepest descent algorithm.^50^ Then the systems were equilibrated sequentially using an *NVT* phase lasting 125 ps with position restraints with a force constant of 400 kJ mol^–1^ applied to the backbone atoms and 40 kJ mol^–1^ applied to the side chain atoms, followed by a 0.002 μs long *NPT* phase, to enable both temperature and pressure to stabilize. The Parinello–Rahman barostat^51^ was used to maintain the pressure at 1 bar with a time constant of τ = 5 ps. A 1 fs integration step was used for equilibration, and a 2 fs integration step was used for the production run.

### Analyses

Visualization and image creation were performed with VMD 1.9.3 ^52^ and Chimera 1.16.^53^ VMD utilizes STRIDE^54^ for secondary structure assignment (e.g., cartoon representation of proteins). Analyses were performed with locally written scripts utilizing the MDAnalysis Python package^55,56^ in multiple instances, MDTraj,^57^ as well as the GROMACS analysis tools. The root-mean-square deviation (RMSD) was calculated on the backbone with the closed structure of RbsB used as a reference.

The salt bridge analysis between key amino acids was carried out due to its importance in protein stability.^58–60^ A cutoff distance of 0.4 nm was applied to define the presence of a salt bridge between two atoms.^61–63^ The distance was measured between the oxygen atom of the anionic carboxylate group of glutamic acid and the nitrogen atom of the cationic ammonium group of the guanidinium group of arginine.

The initial binding site (later used to consider ribose dissociation from the binding site) was defined as all protein atoms within 0.4 nm of the ribose at the start of the production simulation. Distances were subsequently measured from the center of mass of the ribose to this binding site. Water molecule residency times were calculated based on a proximity criterion of ≤0.35 nm from the initial binding site.

The SASA calculation followed the protocol by Alessandri et al.^64^ with 4800 grid points and a probe size of 0.191 nm. The van der Waals radii were taken from the literature, consistent with the values used by Alessandri et al.,^64^ and originally reported by Rowland and Taylor.^65^ Principal component analysis (PCA) was performed with the GROMACS simulation suite on the backbone of the structures. The opening angle was measured between the Cα atoms of Leu37 and Gln160, located in the β-sheets of the two separate main domains of RbsB, and Asp264, positioned in the hinge region connecting the two domains (Figure 1B).

### Convergence of Simulations

Convergence of conformational sampling was assessed using a clustering-based approach. Trajectories from all independent replicas were concatenated into a single ensemble for each system and FF (5 μs total each; 7 μs for 2DRI holo systems). Structures were clustered using an algorithm credited to Daura et al.^66^ with a 0.2 nm backbone RMSD cutoff. The algorithm iteratively identifies the structure with the largest number of neighbors within the threshold, assigning these frames to a cluster until all frames are eliminated.

Cumulative mean RMSD and the associated standard deviation were evaluated across all replicas for each FF using the concatenated 5 μs trajectories with a cumulative block size of 0.01 μs.

We quantified statistical uncertainty using block analysis of RMSD following the protocol of Grossfield and Zuckerman.^67^

## Results and Discussion

### Apo versus Holo States of the Protein RbsB

We first analyzed our simulations of a single structure of RbsB (2DRI, resolution 1.6 Å) in its apo and holo states with all three FFs. The secondary structure of the protein was stable in all apo and holo simulations as expected (see Figure 2) as evidenced by root-mean-square deviation (RMSD) from the initial structure and secondary structure analyses (Figure S1). In all simulations of the apo protein, the RMSD had reached a plateau around ∼0.51 nm by the end of the simulation. There was greater variation in the values for the ribose-bound protein systems. Visual inspection of the trajectories revealed differences in both the ribose residence times within the binding site and protein conformational dynamics. If we consider the ribose residence times first; specifically, in the AMBER and CHARMM simulations, ribose disassociated from the protein binding site in only one replica (per force field); in contrast, dissociation occurred in four replicas of the SIRAH hybrid simulations (see Figure 2).

**Figure 2:**
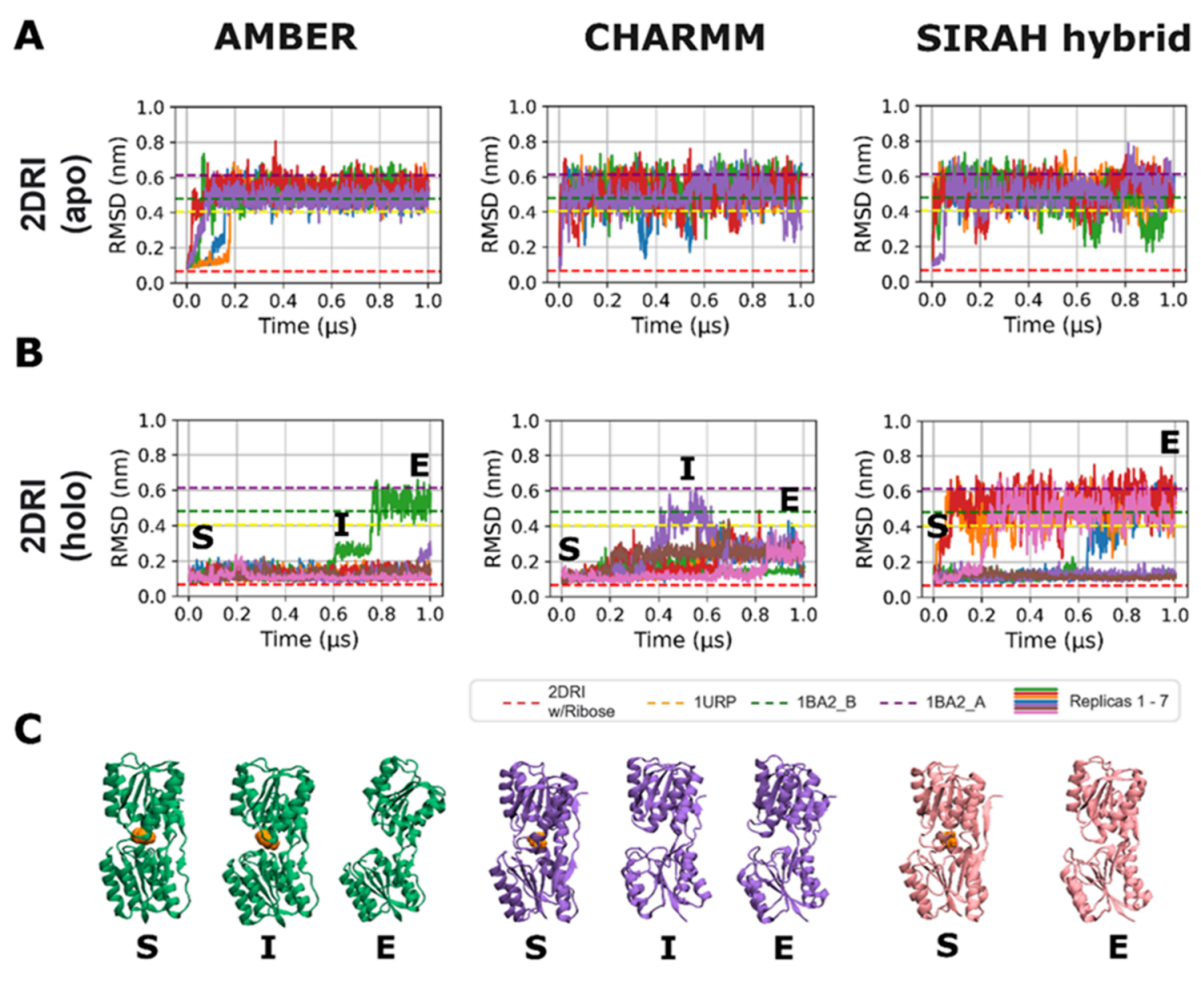
(A) RMSD over time of the two initial closed configurations, 2DRI with (holo) and without (apo) ribose. The RMSD values corresponding to the X-ray structures of the protein in an open conformation are indicated by the yellow, purple, and green dashed lines (PDB code 1URP and PDB code 1BA2, structures A and B, respectively) and closed conformation by the red dashed line (PDB code 2DRI). (B) The ribose-bound 2DRI, showed two distinct scenarios; (i) ribose remained in the binding site, and in these simulations, the protein remained closed, with RMSD values characteristic of the closed conformation (red dashed line) and (ii) ribose left the binding site and in these cases, the protein conformation reached RMSD values typical of the open conformation (see dashed lines). (C) The opening process in selected replicas where ribose left the binding site. Conformation “S” represents the starting structure, while conformation “E” corresponds to the final structure and “I” marks an intermediate conformation.

Additionally, RMSD time series were analyzed across all replicas to explicitly assess replica-to-replica variability and the contribution of ribose dissociation events to apparent FF-dependent behaviors (Figure S2). In the apo 2DRI simulations, RMSD profiles were comparable across FFs, reaching stable plateaus with relatively narrow standard deviations, indicating consistent sampling of similar conformational ensembles across replicas. In contrast, the holo 2DRI simulations exhibited comparably larger RMSD variability when all replicas were included. This increased variance coincided with trajectories in which ribose dissociated from the binding site. When replicas exhibiting ribose dissociation were excluded, the mean RMSD values decreased, and the standard deviation narrowed markedly for all FFs. This shows that large RMSD excursions in the holo simulations were primarily driven by conformational changes from discrete ribose dissociation events. Notably, FF-dependent differences in RMSD plateaus persisted even after excluding dissociating replicas.

Taken together, these results suggest that observed FF trends reflect differences in the frequency with which specific conformational transitions are accessed, but do not reflect uniform shifts in the underlying conformational landscape.

Now we turn to the conformational variations – specifically the angle, defined between the two domains of RbsB and hinges (Leu37, Gln160, and Asp262; Figure 1B). In most cases, this angle from the simulations reproduced the values associated with either the open or closed conformations of RbsB calculated from the X-ray structures of 1URP and 2DRI, respectively (Figure 3A)—an in-depth discussion follows. Ribose dissociation was associated with an opening angle consistent with that of the wt open conformation (∼94°), except in the CHARMM system, where persistent salt-bridges restricted full opening (see below for additional detail) despite ribose dissociation.

**Figure 3:**
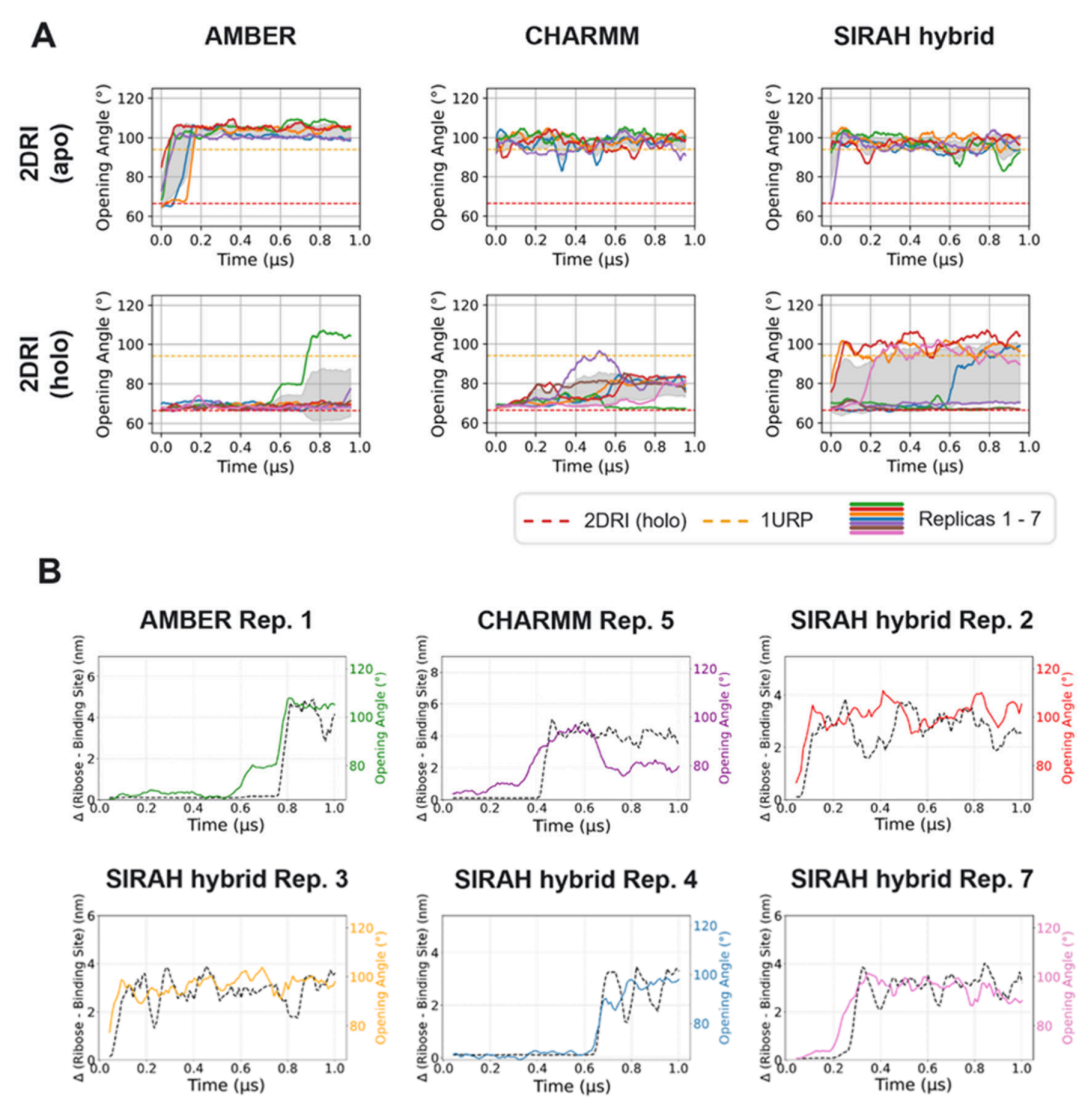
All data are shown as a running average over a 0.05 μs window. (A) Opening angle over time for simulations starting from the closed 2DRI state, with and without ribose, for the AMBER, CHARMM and SIRAH hybrid FFs. In several replicas (one AMBER and CHARMM simulation each; four SIRAH hybrid simulations), the protein transitioned to an open state. The opening angle corresponding to the wt X-ray structure of the protein in open conformation is indicated by the yellow dashed line (PDB code 1URP) and closed conformation by the red dashed line (PDB code 2DRI). (B) Distance between ribose and the initial ribose binding site versus the protein opening angle over time for replicas where ribose dissociated. These plots show that protein opening generally precedes the departure of ribose.

To determine the sequence of events, whether a conformational change in the protein led to ribose dissociation and exit from its binding site, or whether the departure of the ribose triggered the conformational change, we monitored both the distance between the ribose and its binding site and the variations in the opening angle as a function of time (see Figure 3B). These data revealed that the conformational change occurred first—except SIRAH hybrid replica 4, where the two events occurred simultaneously—subsequently leading to the release of the ribose from the binding site.

We next analyzed the nature of the ribose-protein interactions, including the key residues directly involved in the conformational transition of RbsB.

### Role of the Ribose Binding Site in Maintaining the Global Structure of RbsB

The ribose binding site contains four residues charged at physiological pH that contact the ribose in the X-ray structure: Asp89 and Arg90 in one domain, and Arg141 and Asp215 in the other (Figure 4A, inset). In the simulations, these residues were observed to form internal (intradomain) salt bridges (Asp89–Arg90 and Arg141–Asp215), with occasional interdomain interactions, particularly between Asp89 and Arg141. As shown in Table S1, interestingly, the only salt bridge present in the X-ray structure is between Asp89 and Arg90.

**Figure 4:**
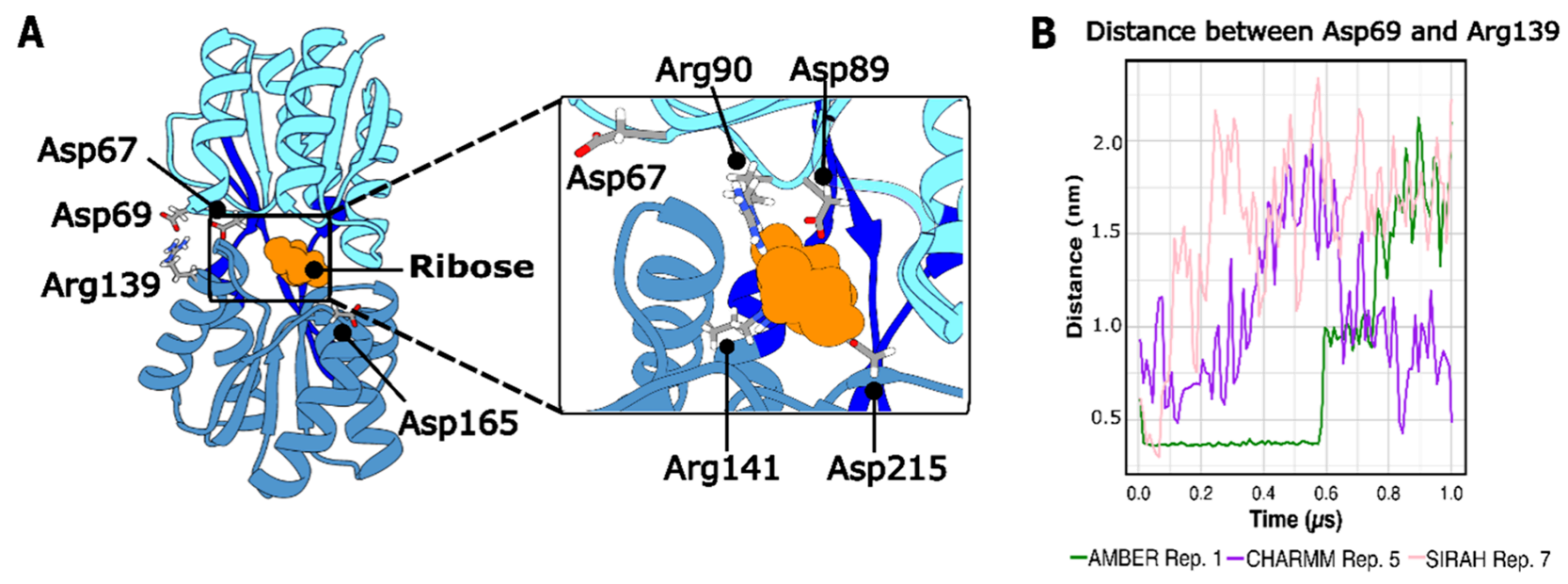
Principal interactions in and around the RbsB ribose binding site. (A) The closed, ribose-bound state (PDB code 2DRI). Ribose is shown in orange, the N-terminal domain in cyan, the C-terminal domain in blue, and hinge regions in dark blue. Key salt bridge residues are highlighted. The inset shows a detailed view of the binding site residues. (B) Distance over time between salt bridge residues Asp69 and Arg139. Data are shown for representative AMBER (green), CHARMM (purple), and SIRAH hybrid (pink) simulations where the protein transitioned to an open state.

Analyses of the salt bridge, RMSD, and angle openings as a function of time revealed an additional important salt bridge between Asp69 and Arg139, located not in the ribose binding site but in a location opposite the hinges (see Figure 4A). This salt bridge was previously reported by Björkman et al.,^19^ but it was only observed when Gly134 was mutated to Arg. It was not detected in wt configurations, leading to the conclusion that the mutation enabled a conformational change necessary for the formation of the Asp69–Arg139 salt bridge in the mutated structure. However, given that we found that the salt bridge was also present in the wt protein, we suggest that such a conformational change may not be strictly required for formation of the Asp69–Arg139 salt bridge. Below, we discuss the correlations between these metrics in more detail.

When ribose remained bound within its binding site, and the protein maintained a closed conformation, the salt bridge between Asp69 and Arg139 was preserved (Figure S3), except for the system described with CHARMM, in which the protein explored more open conformations (Figure 3A). Conversely, ribose dissociation coincided with increased distances between Asp69 and Arg139 (see Figures 2 and 4B). This suggests that the salt bridge between Asp69 and Arg139 may stabilize the closed conformation. Below, we take a more detailed look at individual system dynamics as a function of FF.

In AMBER replica 1, the ribose remained bound at ∼0.7 μs (see Figure 2C, intermediate conformation, “I”), but the Asp69–Arg139 salt bridge was lost, leading to a more open conformation of RbsB (Figures 2 and S3). This resulted in the loss of the remaining internal salt bridges to ribose at ∼0.76 μs, followed by the ribose leaving the binding pocket. Thus, the Asp69–Arg139 salt bridge was lost prior to ribose dissociation.

In one of the CHARMM replicas (number 5), the formation of a new salt bridge (not present in the X-ray structure, Table S1) between residues Asp89 and Arg141, which are in different domains (Figure 4A inset and Figure S4), introduced a conformational change that destabilized the Asp69–Arg139 salt bridge, resulting in a more open conformation (see Figures 3A and 4). Interestingly, after the ribose left the binding site, the transient formation of another intradomain salt bridge between Arg141 and Asp215 took place (Figure S4). In replica 1, in which the protein remained in a closed conformation (Figure 2); the Asp69–Arg139 salt bridge broke at a very early stage (∼0.1 μs) (Figure S3). Upon closer inspection, a new interdomain salt bridge between Asp89 and Arg141 was formed after ∼0.3 μs (see Figure S5). In this simulation, it is likely that this interaction helped maintain the protein in a closed conformation despite the loss of the Asp69–Arg139 salt bridge.

In SIRAH hybrid replica 7, the ribose first lost its interaction with the binding site residues and transiently formed hydrogen bonds with Asp67 and subsequently Asp69, before ultimately losing all contact with the protein. In replicas 2 and 3, by contrast, the ribose dissociated from the binding site at an early stage (∼0.05 μs). The Asp69–Arg139 salt bridge was never formed (see Figures 2 and S3) and RbsB maintained an open conformation.

In SIRAH hybrid replica 4, the Asp69–Arg139 salt bridge was first broken at ∼0.07 μs (see Figure S3). However, the salt bridges at the ribose binding site rearranged, resulting in the formation of an interdomain interaction between Asp89 and Arg141, similar to one observed in the CHARMM scenarios. This interaction was lost at ∼0.6 μs, at which point the protein also began transitioning to an open conformation (Figures 3A, S3, and S6), which allowed the ribose to leave the binding site.

We next examined the presence of any water molecules that may mediate interactions between the ribose and the surrounding amino acids in the binding site. In CHARMM replica 5 and SIRAH hybrid replica 4, a conserved water molecule was identified, persisting for ∼0.41 μs and ∼0.44 μs, respectively. In the AMBER replica 1 simulation, two water molecules were observed occupying the same position sequentially, with residency times of ∼0.48 and ∼0.31 μs, yielding a combined presence of ∼0.79 μs. A structurally equivalent water molecule was also observed in the 2DRI crystal structure, providing further supporting that it can play a role in mediating the ribose-protein interaction.

In all cases, the water molecule formed hydrogen bonds with the ribose as well as residues Arg89 and Asp141 of RbsB (see Figure S7). Notably, we previously observed Arg89 and Asp141 to form a salt bridge associated with the conformational transition of RbsB from closed to open (see Figure 4). As this water molecule was also present in systems where the ribose remained attached to the binding site, we propose that this water molecule contributed to ribose stabilization within the binding site.

In summary, the simulations of 2DRI revealed a detailed, albeit FF-dependent, mechanism for the dissociation of ribose from RbsB. The primary event initiating the release of ribose was a conformational change in the protein from a closed to an open state. This structural transition was linked to the stability of a key salt bridge between Asp69 and Arg139. The disruption of this salt bridge coincided with the opening of the protein and the subsequent departure of ribose. The simulations also highlighted the importance of a conserved water molecule, observed in the crystal structure (PDB code 2DRI) as well, which may help to stabilize the bound ribose by mediating hydrogen bonds with key residues in the binding site.

### Investigation of Force-Field-Dependent Behavior

Given that we observed FF-dependent ribose residence times within the binding pocket of RbsB, it was pertinent to conduct a more thorough investigation of FF-dependent behaviors of the apo RbsB structures: thus, we simulated the wt (PDB code 1URP) and AlphaFold structure (AF-P02925). To enable comparison with the published work of Björkman and Mowbray^2^ two apo mutant structures (PDB code 1BA2, which contains two structures, A and B) were also simulated. Björkman and Mowbray^2^ previously described these mutant conformations as hyperextended given their wide opening angles (∼113 and ∼100° for A and B, respectively, compared to ∼94° for 1URP, the open wt structure).

Each of the six starting structures was simulated for at least 5 × 1 μs, with AMBER, CHARMM, and SIRAH hybrid force fields. The RMSD from the starting structure was first measured to evaluate the structural drift. The RMSD of all apo RbsB structures showed no systematic drift during the simulations, maintaining plateau values of ∼0.65 nm (1BA2_A and 1BA2_B) and ∼0.51 nm (1URP, 2DRI, and AF-P02925) for all three FFs (see Figures 2 and S8). The simulations of 2DRI with ribose can be divided into two groups, one group of replicas which fluctuated around an initial value of ∼0.15 nm and another group in which the RMSD showed greater fluctuations (Figure 2). The latter group coincided with instances where the ribose dissociated from the binding site, whereas the lower fluctuation group retained ribose binding throughout the simulation time.

Across all systems, the AMBER simulations generally exhibited smaller RMSD fluctuations than the CHARMM and SIRAH hybrid FF simulations. It is possible that the improved dipole moment and dielectric properties of OPC^24,68^ may have contributed to more accurate water–protein interactions in the AMBER simulations. Interestingly, despite the CHARMM and SIRAH hybrid systems differing in their bulk water models (due to the addition of WT4 in the SIRAH hybrid systems), the time-dependent fluctuations as well as the final RMSD values were similar.

Next, we compared the radius of gyration (RoG) density distributions to assess the compactness of RbsB across simulations (Figure 5). All of the RoG values calculated from the experimental X-ray structures were sampled in our simulations (Figure 5). The RoG values distributions of the AMBER simulations of 1BA2_A, 1BA2_B, 1URP, and 2DRI without ribose aligned closely with the RoG of the mutant B structure, peaking near ∼2.05 nm (Figure 5). CHARMM and SIRAH hybrid simulations of the same structures exhibited broader, left-shifted distributions with lower peaks, indicating more compact states compared to the AMBER simulations (Figure 5).

**Figure 5:**
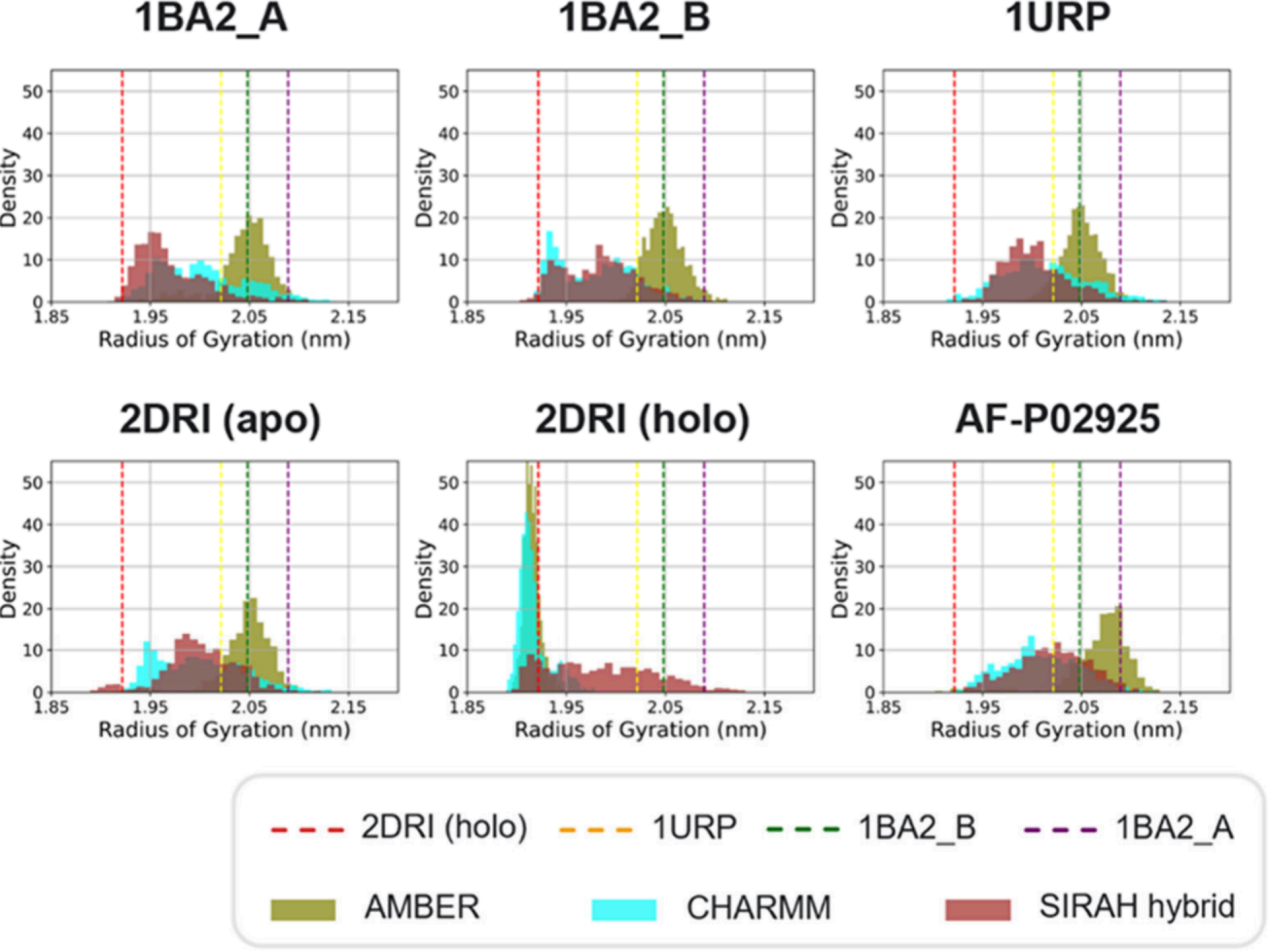
Density distributions of the radius of gyration (RoG) for different starting structures and FFs, combining data from all replicas. The RoG values corresponding to the X-ray structures of the protein in an open conformation are indicated by the yellow, purple and green dashed lines (PDB code 1URP and PDB code 1BA2, structures A and B, respectively) and closed conformation by the red dashed line (PDB code 2DRI). The RoG distributions highlight FF-dependent behaviors; AMBER simulations resulted in a less compact apo state on average compared to CHARMM and SIRAH hybrid, while SIRAH hybrid simulations showed a broader conformational spread for the closed holo state.

All FFs sampled the RoG value calculated from the 2DRI bound-state structure (Figures 5 and S9). In the simulations during which ribose dissociated, the SIRAH hybrid simulations had a broader distribution of RoG values compared to the AMBER and CHARMM simulations. This is consistent with differences in the ribose residency times described earlier (compare with Figure 2).

We next examined the RoG values characteristic of the conformations of hyperextended mutants 1BA2–A (∼2.09 nm) and 1BA2_B (∼2.05 nm). Björkman and Mowbray^2^ had suggested that these conformations may be crystallographic artifacts representing a less prevalent solution state compared to the wt. We were able to reproduce both mutant RoG values with all the FFs for all starting apo structures, including the wt (Figure S9), and for the ribose-bound structures after ribose had dissociated. However, only the AMBER simulations of the AF-P02925 wt system yielded a RoG distribution centered around the value calculated for mutant A (Figure 5), indicating that with AMBER, this level of compactness was maintained rather than being transiently visited.

While TIP3P may have been expected to yield larger RoG values due to weaker stabilization of surface residue interactions, and OPC to favor more compact structures due to improved modeling of water–protein interactions,^24,69^ we observed that AMBER (with OPC water) produced more discrete RoG distributions, matching mutant B in the open state and 2DRI with ribose in the closed state. Conversely, the SIRAH hybrid system explored a wider range of RoG values, perhaps due to reduced friction from the coarse-grained WT4 solvent. This dynamic behavior correlates with the fact that in four out of seven replicas, ribose was lost and the protein transitioned from a closed to an open conformation.

To investigate the open-close conformational transition of RbsB, we calculated the time-dependent opening angle (see Figures 6 and S10 for full plots) and solvent-accessible surface area (SASA) throughout the simulations (Figure S11).

**Figure 6:**
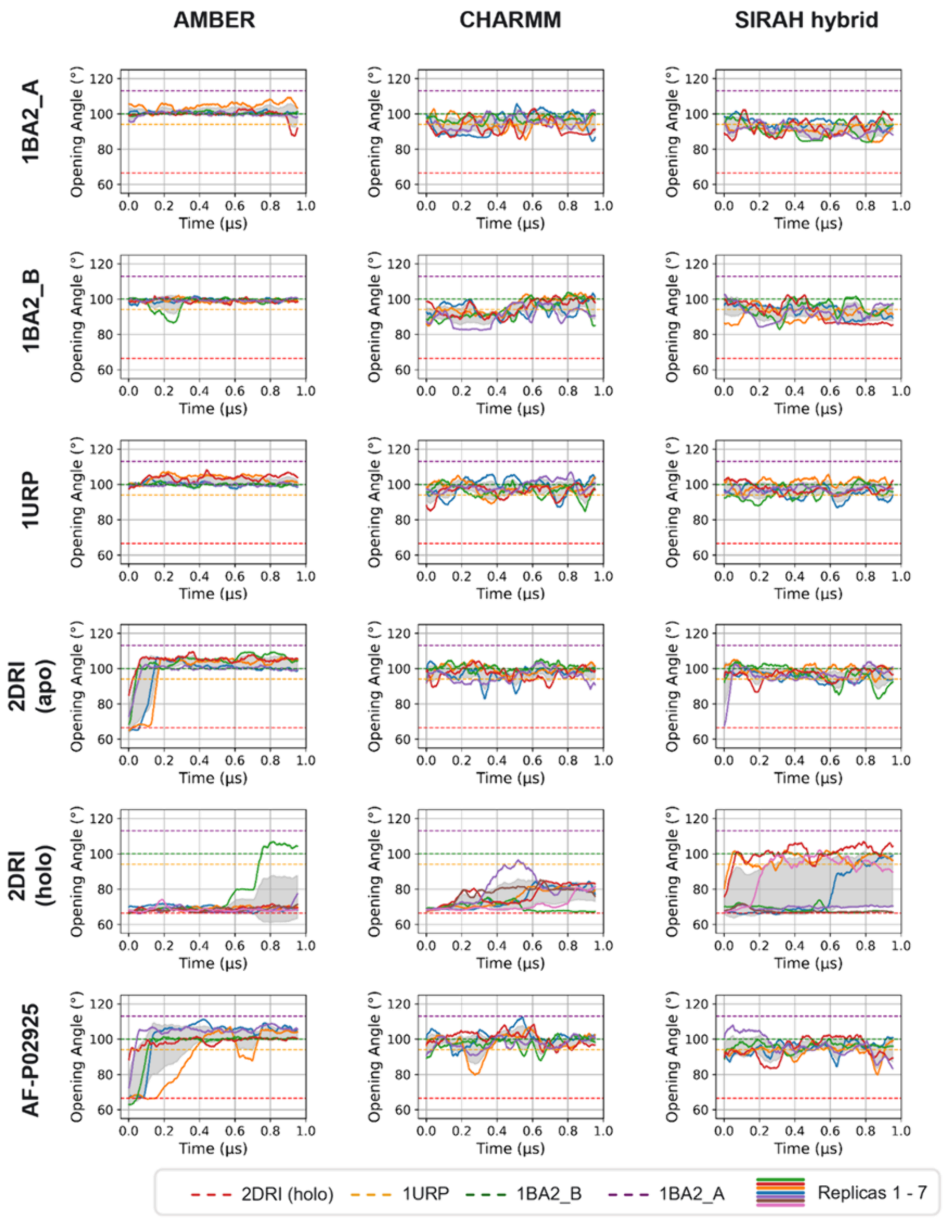
Opening angle over time for all simulated systems, shown as a running average (0.05 μs window size) with standard deviation shaded in gray. The opening angle values corresponding to the X-ray structures of the protein in an open conformation are indicated by the yellow, purple, and green dashed lines (PDB code 1URP and PDB code 1BA2, structures A and B, respectively) and closed conformation by the red dashed line (PDB code 2DRI). Simulations of ribose-bound 2DRI showed three distinct states: a closed state resembling ligand-bound 2DRI at 66.4°, an intermediate state fluctuating around ∼80.0°, and an open state consistently exceeding ∼90.0°. This fully open state was observed in one replica each for AMBER and CHARMM and in four replicas for the SIRAH hybrid FF.

Excluding simulations with ribose, we observed that in the AMBER FF, the opening angle of RbsB stabilized at ∼100°, which is similar to the value reported for the structure, 1BA2_B (Figure 6 and Table 2). In contrast, in simulations with the CHARMM and the SIRAH hybrid FFs the angle fluctuated around a value of ∼94.0°, which is closer to the opening angle of 1URP. Despite these differences in mean behavior, the overlapping standard deviations across AMBER, CHARMM, and SIRAH hybrid simulations suggested trends rather than statistically distinct behaviors. Notably, CHARMM and SIRAH hybrid simulations showed greater fluctuations (∼6.6°) than AMBER (∼4.9°) (Table 2).

**Table 2:** Average Opening Angles and Standard Deviation^a^

	AMBER	CHARMM	SIRAH hybrid
1BA2_A	101.1° ± 5.0°	95.8° ± 6.9°	91.3° ± 6.0°
1BA2_B	99.2° ± 4.0°	95.7° ± 7.1°	92.4° ± 6.5°
1URP	101.4° ± 4.7°	97.7° ± 6.9°	97.2° ± 6.0°
2DRI (apo)	103.2° ± 4.8°	97.9° ± 6.6°	96.2° ± 6.4°
AF-P02925	102.5° ± 5.9°	95.1° ± 6.6°	95.1° ± 6.6°

a Values were calculated from the final 0.6 μs of the production run, averaged across all replicas. The table includes data for systems starting from open (1BA2_A, 1BA2_B, and 1URP) and closed (apo 2DRI and AF-P02925) conformations.

Additionally, in AMBER simulations of 2DRI and AF-P02925, the opening angles sometimes took over 0.1 μs to stabilize—a delay not observed in other FFs (Figure 6). The SASA profiles supported these findings, with a larger solvent-exposed area corresponding to open protein conformations (Figure S11).

In simulations of 2DRI with ribose, we identified three distinct opening angle states (Figure 6): a closed state resembling ligand-bound 2DRI at 66.4°, an intermediate state fluctuating around an opening angle of ∼80.0°, and an open state consistently exceeding ∼90.0°. Among the seven replicas per FF, one AMBER and one CHARMM simulation exhibited the fully open state, while four SIRAH hybrid FF simulations reached this conformation. The intermediate state could be observed in AMBER and CHARMM simulations (Figure 6).

The time-dependent values of the binding site-ribose distance and the opening angle of RbsB (see Figure 3B) confirmed that an opening angle exceeding ∼90.0° correlated with ribose leaving the 2DRI binding pocket, whereas for fluctuations around ∼80.0° the ribose did not leave the binding site. No instances of ribose rebinding were observed after dissociation.

Among all simulations, in only one did the protein achieve the opening angle observed in the X-ray structure of the hyperextended mutant 1BA2_A (113.1°) for at least 0.05 μs: a CHARMM simulation of the AF-P02925 structure, which reached an opening angle of 114.8° around ∼0.56 μs (Figure 6, bottom panel).

We next performed principal component analysis (PCA) to assess large-scale motions across the starting structures. Eigenvalues were derived from a SIRAH hybrid replica that sampled both closed and open states, and we acknowledge a potential bias toward the principal components of the FF. The eigenvalues explaining 89.9% of the total variance. The resulting eigenvectors were used to project all other 1 μs replicas across FFs into the same PCA space to allow quantitative comparison across FFs (Figure 7).

**Figure 7:**
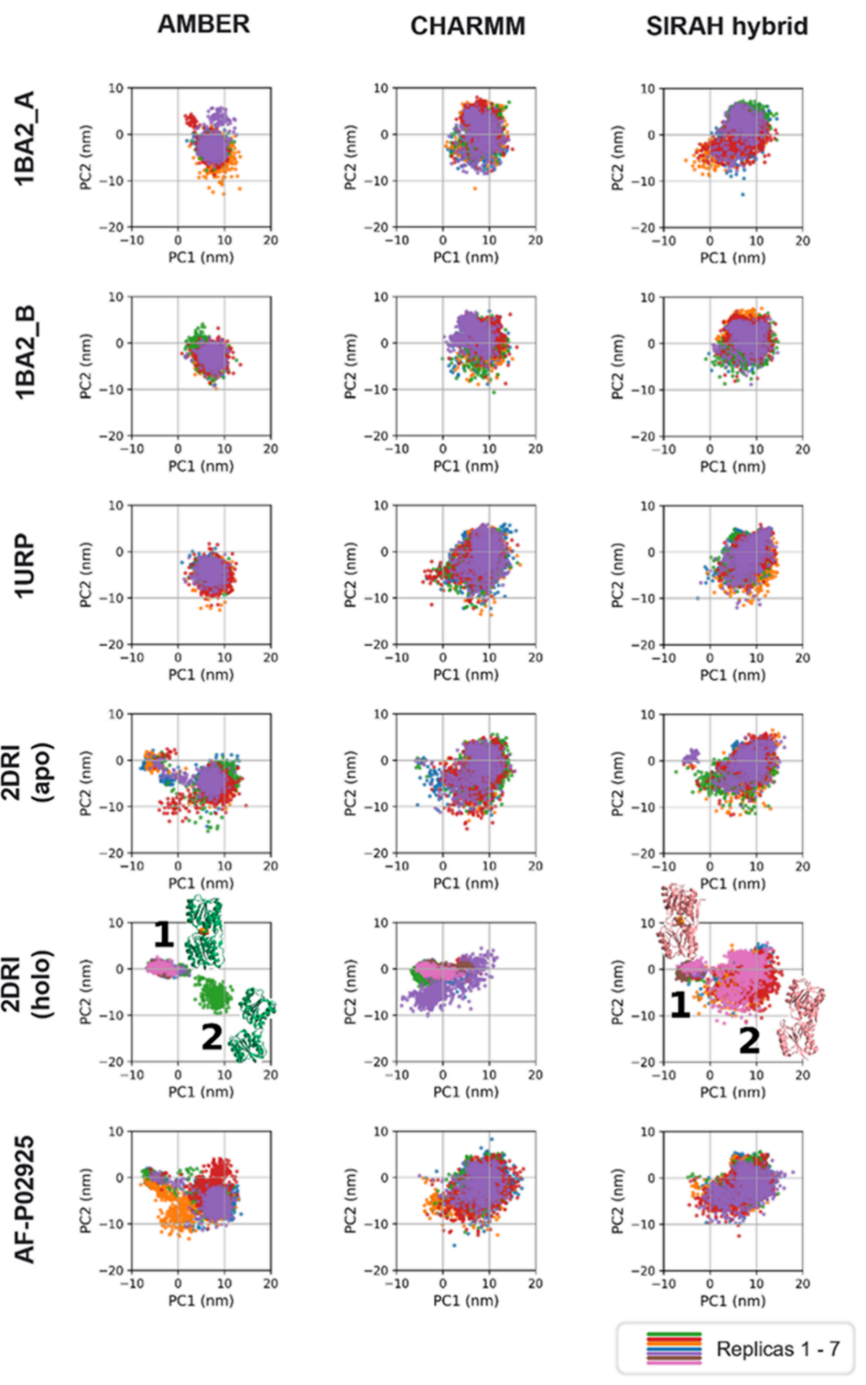
Principal component analysis of all simulated trajectories. Simulations of the ribose-bound 2DRI system using the AMBER and SIRAH hybrid FFs exhibited two distinct clusters (“1” and “2”) corresponding to the closed holo and open apo states. Representative conformations are depicted. In contrast, simulations starting from open conformations (PDB code 1URP and PDB code 1BA2, structures A and B, respectively) formed a single, open-state cluster.

We were able to distinguish two clusters in the 2DRI-with-ribose simulations using the AMBER and SIRAH hybrid FFs (Figure 7, denoted as clusters “1” and “2”, respectively). The opening angle of the structures confirmed that cluster “1” represented the closed, holo conformation, while cluster “2” represented the open, apo state. In contrast, the CHARMM simulations of 2DRI with ribose formed a single cluster. This likely reflected the opening angle dynamics: only one of the seven CHARMM replicas transitioned to the open conformation to release the ribose before returning to an intermediate state (see Figure 6). The oval shape of the cluster likely arose from six of the seven replicas remaining in this intermediate state—a behavior not observed with the SIRAH hybrid FF and seen only briefly at the end of one of the AMBER simulations.

PCA revealed that the formation of the closed-conformation cluster “1” could only be partially achieved by delayed opening dynamics at the start of the simulations: cluster “1” began to emerge in replicas of 2DRI without ribose, as well as for AF-P02925 (compare Figure 6). PCA of the three open starting structures (1BA2_A, 1BA2_B, and 1URP) revealed for each a single cluster corresponding to the open conformation, as expected. However, simulations using the AMBER FF sampled a more confined region of the PCA space compared to those with CHARMM and SIRAH hybrid FFs, suggesting reduced exploration of large-scale motions (Figure 7).

To establish a robust assessment of convergence and coverage of the conformational phase space of the systems we have used three convergence analyses: (i) clustering-based conformational analysis, (ii) cumulative mean RMSD with standard deviation stabilization, and (iii) block-averaging to quantify uncertainty and sampling quality following Grossfield and Zuckerman.^67^

First, convergence of conformational sampling was assessed using a clustering-based approach. Trajectories from all independent replicas were concatenated into a single ensemble for each system and FF (5 μs total each; 7 μs for 2DRI holo systems) and clustered. The most populated clusters contained frames originating from multiple independent replicas rather than from a single trajectory (Figure S12). This inter-replica mixing suggests that separate simulations converged to the same dominant conformational basins rather than remaining trapped in replica-specific local minima, indicating effective exploration of the accessible phase space within 1 μs per replica.

Second, cumulative mean RMSD and the associated standard deviation were evaluated across all replicas for each FF using the concatenated 5 μs trajectories (Figure S13). This analysis was performed on the apo 2DRI simulations, given that we observed little to no conformational change until ribose dissociation. The cumulative mean RMSD rapidly approached a plateau and remained stable thereafter, indicating the absence of a systematic drift. In parallel, the standard deviation converged to a time-independent band, demonstrating that the amplitude of structural fluctuations stabilized over the simulated time frame. Together, these observations indicate that the RMSD observable reached statistical stationarity over the sampled time scale.

Third, we quantified statistical uncertainty using block analysis of RMSD following the protocol of Grossfield and Zuckerman.^67^ All replicas and FFs of the 2DRI holo simulations were combined into a single 21 μs trajectory, enabling assessment of whether both closed and open conformational basins, as well as intermediate states, were sufficiently sampled in this study. The estimated standard error converged with increasing block size, reaching a maximum global uncertainty of below 0.3 nm (Figure S14). Saturation of the uncertainty with block size indicates that additional sampling would not substantially change the ensemble-averaged RMSD, supporting sufficient sampling of the relevant conformational states.

Taken together, this study includes the three most important steps in ensuring sufficient sampling according to Grossfield and Zuckerman:^67^ the use of (i) independent starting conformations, (ii) the metric of block averaging to estimate statistical uncertainty, as well as visual analyses, and (iii) PCA analysis.

## Conclusion

In this study, we observed the full conformational transition of the periplasmic binding protein RbsB for the first time. Our study revealed that in most scenarios (all but one) the conformational changes precede the departure of ribose from the binding site, a process likely influenced by specific interactions at the binding interface. Indeed, our analyses of ribose binding revealed that specific salt bridges played a crucial role in stabilizing the closed conformation of RbsB. The native salt bridge between Asp69 and Arg139 was essential for maintaining the compact state. We were also able to identify a putative structural water molecule—also present in the crystal PDB structure—that coordinated hydrogen bonds between the ribose, Asp89, and Arg141, with a potential structural role in maintaining the ribose in its binding site.

We identified distinct roles for interdomain and intradomain salt bridges. Interdomain salt bridges, such as Asp89–Arg141 and Asp69–Arg139, stabilized the closed conformation by maintaining contacts between the two main domains. Upon ribose-release, these interactions broke as the domains separated. The now-unpaired Arg141, carrying a positive charge, formed transient intradomain salt bridges (e.g., with Asp215) within the same domain. Together, these observations suggested the presence of a distributed and partially redundant salt-bridge network, in which alternative inter- and intradomain interactions could transiently stabilize closed or intermediate conformations and partially compensate for the disruption of the Asp69–Arg139 interaction.

In addition, our findings refine the suggestion by Björkman and Mowbray^2^ that the hyperextended conformations A and B (PDB code 1BA2) may be crystallographic artifacts representing solution states less prevalent than that of the wt. Indeed, our simulations of the wt protein repeatedly captured an opening angle of ∼100° across all three FFs, in agreement with the mutant B conformation. In contrast, the ∼113° angle characteristic of mutant A was only exceeded in a single simulation (AF-P02925 with CHARMM), suggesting a less common state.

Furthermore, we analyzed the structural dynamics of RbsB by comparing the performance of three force fields; AMBER, CHARMM, and SIRAH hybrid using key structural observables, including RMSD, RoG, interdomain opening angle, and SASA. While all three FFs successfully reproduced the experimentally observed open and closed conformations depending on the starting structure, only closed starting conformations transitioned to the open state, whereas open structures remained open throughout the simulations. The CHARMM and SIRAH hybrid FFs, other than for 1BA2_A, exhibited greater fluctuations in key structural metrics compared to AMBER. Notably, the SIRAH hybrid FF facilitated more frequent and earlier conformational transitions from closed to the open state in the presence of ribose. This may be due to differences in water models, given that the protein is described by the same CHARMM36­(m) parameters, but the bulk water molecules are described using the WT4 in the SIRAH hybrid regime, whereas all waters are described with the TIP3P model in the CHARMM simulations. While we have captured closed to open conformational transitions here, it is important to note that, as with the vast majority of *in silico* and *in vitro* studies of protein conformational dynamics, the systems we have simulated here represent a far more dilute environment than the one that RbsB would naturally encounter *in vivo* within the crowded periplasmic environment of *E. coli*. Thus, simulations of more crowded environments would be a useful future extension for a more detailed examination of the time scales of conformational transitions.

Taken together, our results support a view of ligand release that extends beyond a simple bound–unbound description. Release is enabled by the transient exploration of alternative conformational states accessed through collective interdomain rearrangements, as previously reported for human lysozyme.^70^ These conformations weaken the specific interactions present in the bound state by replacing them with short-lived, noncanonical contacts, thereby promoting ligand or product dissociation.^70^ Consistent with this picture, metadynamics simulations of the closely related glucose/galactose-binding protein revealed that conformational opening events precede glucose unbinding, highlighting a tight coupling between domain rearrangements and ligand dynamics.^71^

Previously reported free energy calculations for RbsB have established a quantitative thermodynamic framework for the open–closed equilibrium, showing that ligand binding stabilizes the closed conformation, whereas in the absence of ribose the open state is entropically favored and separated by moderate free energy barriers involving intermediate states.^20,72^ In line with these observations, our simulations showed that ribose release from RbsB was generally preceded by conformational opening and involved transient alternative salt-bridge interactions stabilizing intermediate states. Together, these findings support a general mechanism in which ligand release in periplasmic sugar-binding proteins is governed by conformational heterogeneity and transient stabilizing interactions.

In conclusion, our study has enabled us to identify the steps in the closed-to-open conformational transition of RbsB and has highlighted the role of both inter- and intradomain salt bridges, some of which were not previously reported. We have shown that using multiple force fields and starting conformations has enabled us to characterize a wide repertoire of RbsB conformations.

## Supplementary Material



## Notes

Additional analyses, including time-resolved secondary structure analysis, RMSD time series, additional data on specific inter- and intradomain distances versus time, and additional details of ribose dissociation from the protein (PDF)
