# Supplementary material for: Characterizing the Conformational Dynamics of the Ribose Transporter B Protein in Escherichia coli: Enhanced Sampling via Multiple Force Fields
Source: J Chem Theory Comput. 2026 Feb 25;22(5):2156–69. doi: 10.1021/acs.jctc.5c02068 (PMC12980723; doi:10.1021/acs.jctc.5c02068)
Supplement: Supplementary file 1 [file ct5c02068_si_001.pdf]

# Supporting Information: Characterizing the Conformational Dynamics of the Ribose Transporter B Protein in *Escherichia coli:* Enhanced Sampling *via* Multiple Force Fields

Nikolai Juraschko[1,2], Florencia Klein Rocha[1] and Syma Khalid[1]*

[1]Department of Biochemistry, University of Oxford, Oxford, OX1 3QU
[2]Rosalind Franklin Institute, Harwell Science & Innovation Campus, Didcot, OX11 0QX
*Correspondence to syma.khalid@bioch.ox.ac.uk

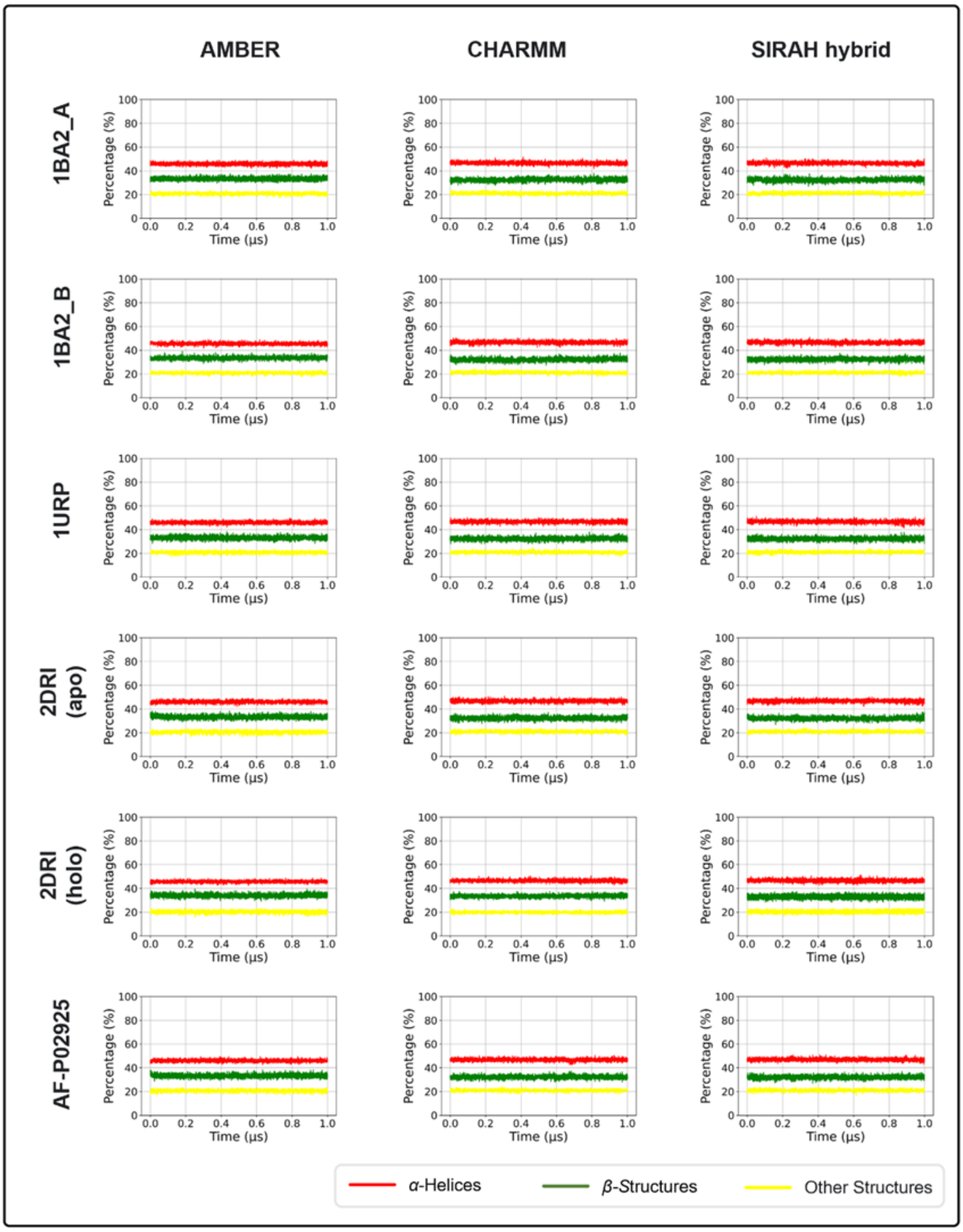


***Figure S1:*** *Time-resolved secondary structure evolution of the six different RbsB structures across the three force fields, AMBER, CHARMM, and SIRAH hybrid, as determined by the DSSP algorithm. The plots depict the percentage of α-helices (red), β-structures (green), and other secondary structure types (yellow) for each of the replicas plotted simultaneously. This figure shows the overall stability of secondary structure content across time, force fields, and structural inputs.*

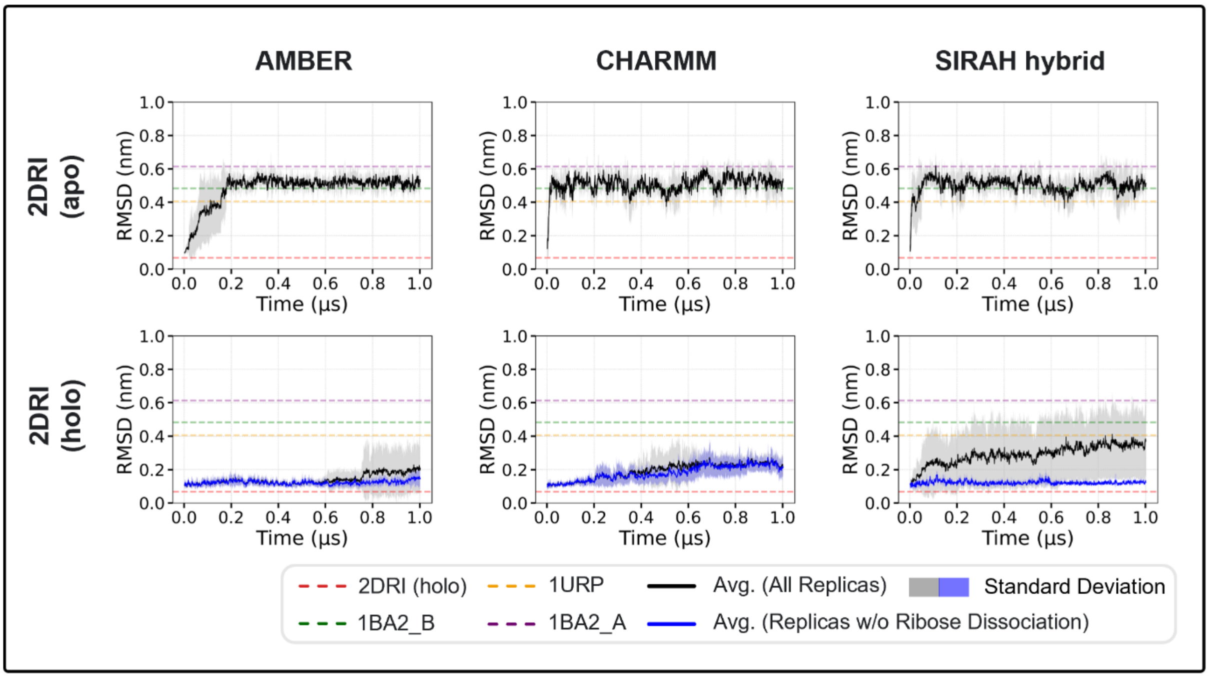


***Figure S2:*** *Backbone root-mean-square deviation (RMSD) time series for the 2DRI systems (apo & holo) simulated with AMBER, CHARMM, and SIRAH hybrid FFs. Solid black lines show the mean RMSD across all replicas, with gray shading indicating the corresponding standard deviation. Solid blue lines and blue shading show the mean RMSD and standard deviation calculated after excluding replicas in which ribose dissociation occurred. The RMSD values corresponding to the X-ray structures of the protein in an open conformation are indicated by the yellow, purple and green dashed lines (pdb codes = 1URP, 1BA2; structures A & B, respectively)[1] and closed conformation by the red dashed line (pdb code = 2DRI).[2] In the apo simulations, RMSD profiles converged to stable plateaus with relatively narrow replica-to-replica variability across all FFs. In contrast, holo simulations displayed increased RMSD variance when all replicas were included. Excluding dissociating replicas reduces both the mean RMSD and the associated variability, indicating that large RMSD excursions primarily reflect conformational changes associated with discrete dissociation events. FF-dependent differences in RMSD plateaus remain evident after removal of dissociation replicas.*

***Table S1:*** *Intra- and interdomain distances between residues in the crystallographic holo structure (pdb code = 2DRI).*

| ***Intradomain Distances*** | |
|---|---|
| *Asp89-Arg90* | *0.37 nm* |
| *Asp215-Arg141* | *0.45 nm* |
| *Asp67-Arg90* | *0.85 nm* |
| ***Interdomain Distances*** | |
| *Asp89-Arg141* | *0.47 nm* |
| *Asp215-Arg90* | *0.85 nm* |
| *Asp69-Arg139* | *0.69 nm* |

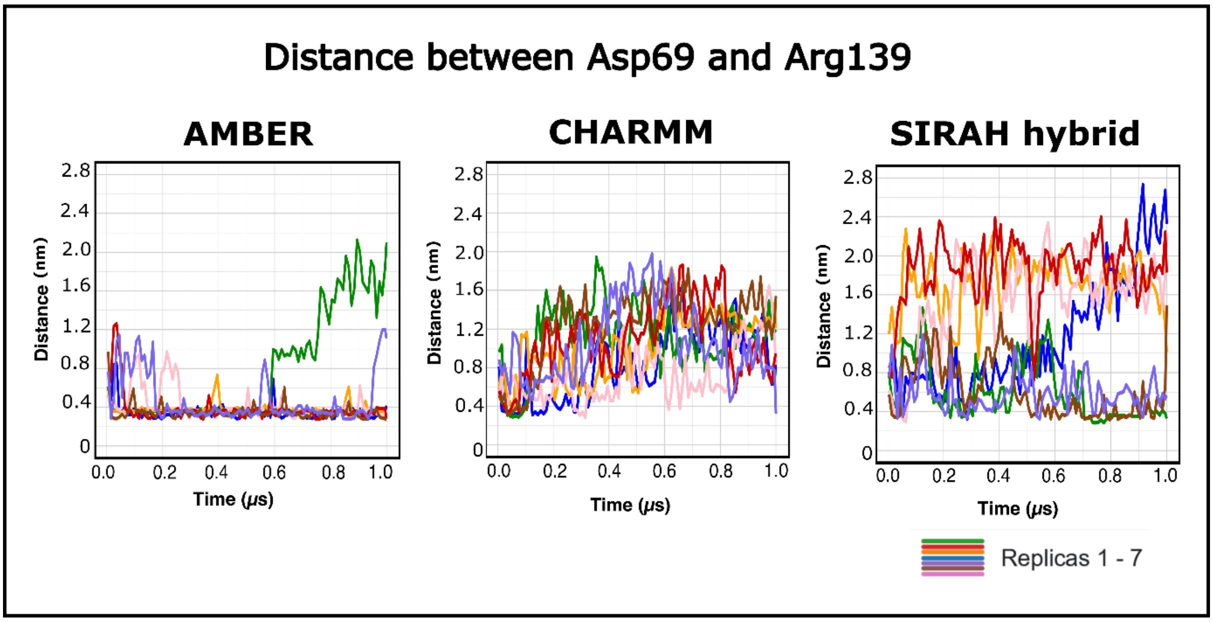


***Figure S3:*** *Distance over time between Asp69 and Arg139 during the simulation for the three FFs. Any small fluctuation in the opening angle or root-mean-square deviation (RMSD) was reflected in the maintenance or loss of this salt bridge (see Figure 2 and Figure 3A). The salt bridge was maintained for the AMBER and SIRAH hybrid FFs whenever the protein remained closed; otherwise, it was lost. Although the ribose dissociated from the binding site only in replica 5 for CHARMM, Figure 3A shows that all replicas adopted a more open conformation, which resulted in the loss of this salt bridge.*

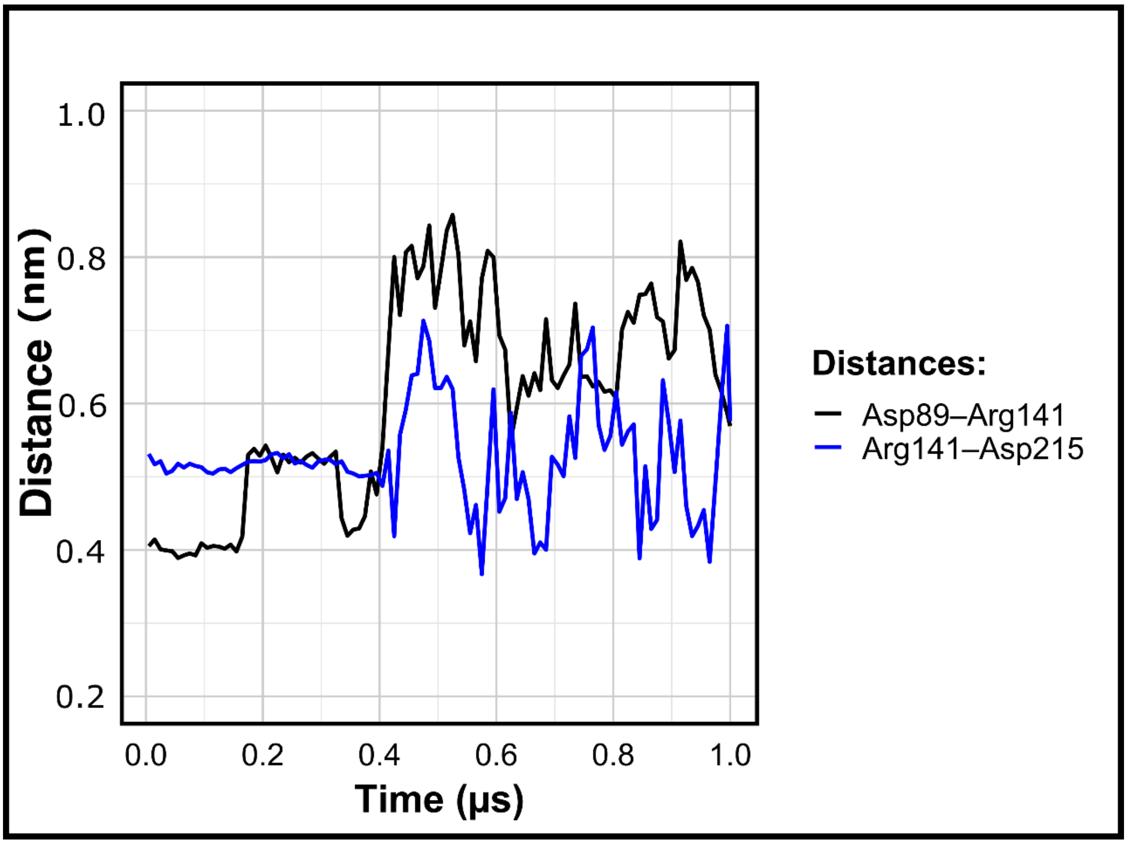


***Figure S4:*** *Salt bridge distances over time in replica 5, CHARMM. The new interdomain salt bridge between Asp89 and Arg141 (black) persisted until ~ 0.17 µs. After its disruption, the protein underwent conformational changes that resulted in the ribose leaving the binding site at ~0.4 µs. This event enabled the transient formation of another new intradomain salt bridge between Arg141 and Asp215 (blue).*

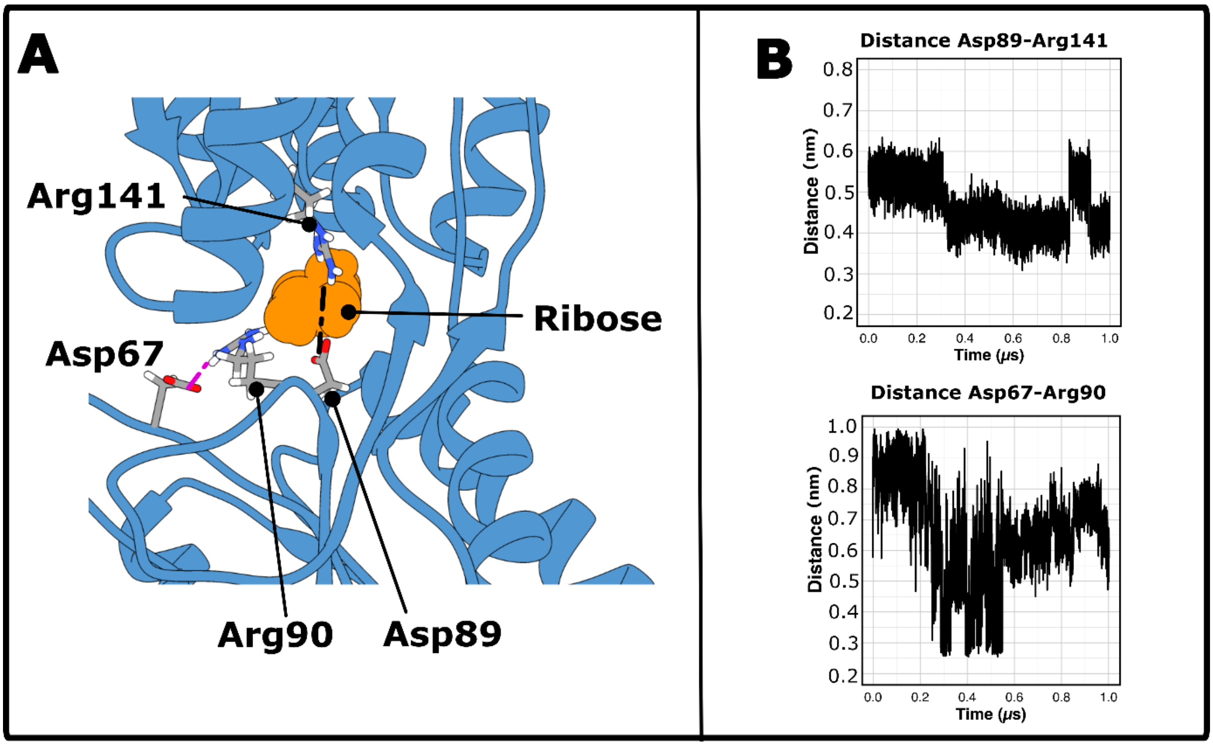


***Figure S5:*** *Panel A: Main interactions in replica 1, CHARMM. The salt bridge between Asp89 and Arg141 is shown as a dashed black line. Another salt bridge formed between Asp67 and Arg90 between 0.25 μs and 0.5 μs (pink dashed line). Panel B: Top: Distance over time between As89 and Arg141. This salt bridge was maintained for most of the simulation, helping stabilize the closed conformation due to its strategic position (see Figure 4). Bottom: Distance over time between Asp67 and Arg90.*

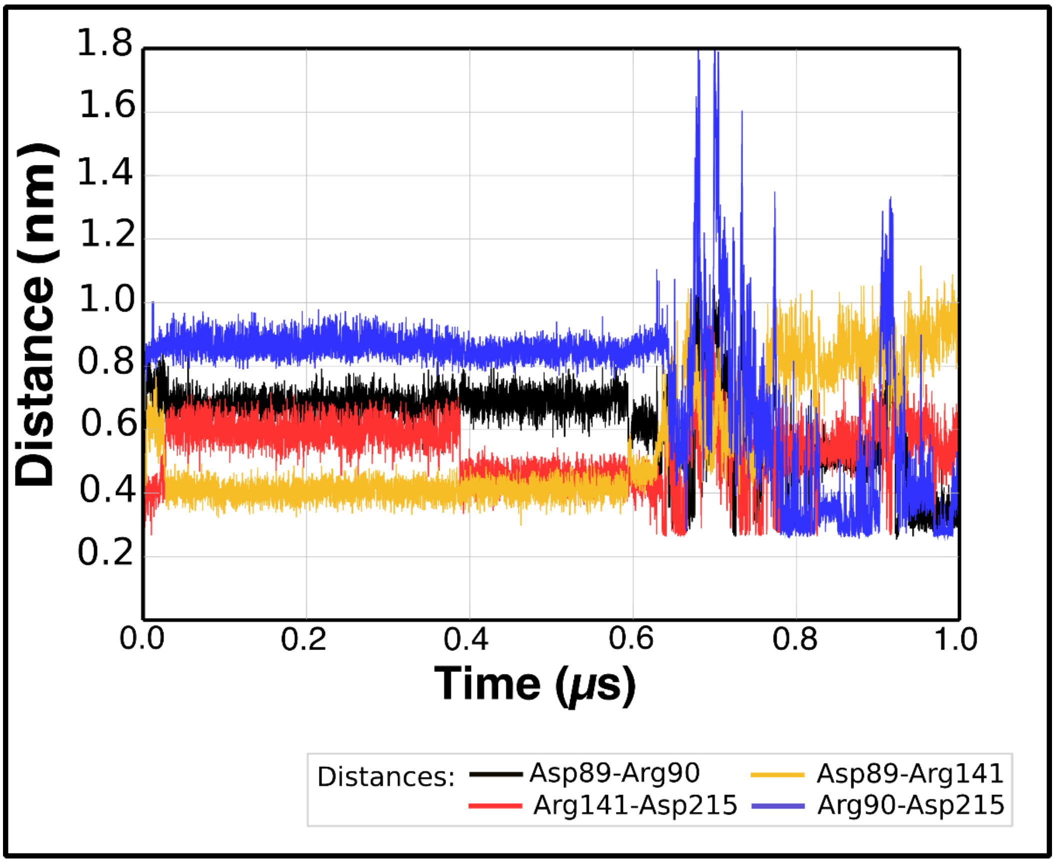


***Figure S6:*** *Distance of internal salt bridges over time in the holo RbsB structure with the SIRAH hybrid FF, replica 4. The interdomain salt bridge between Asp89 and Arg141 (orange) was essential to maintain the closed conformation until ~0.6 μs. A secondary interaction between Arg141 and Asp215 (red) also contributed. Distances between Asp89-Arg90 (black) and Arg90-Asp215 (blue) are also shown.*

.

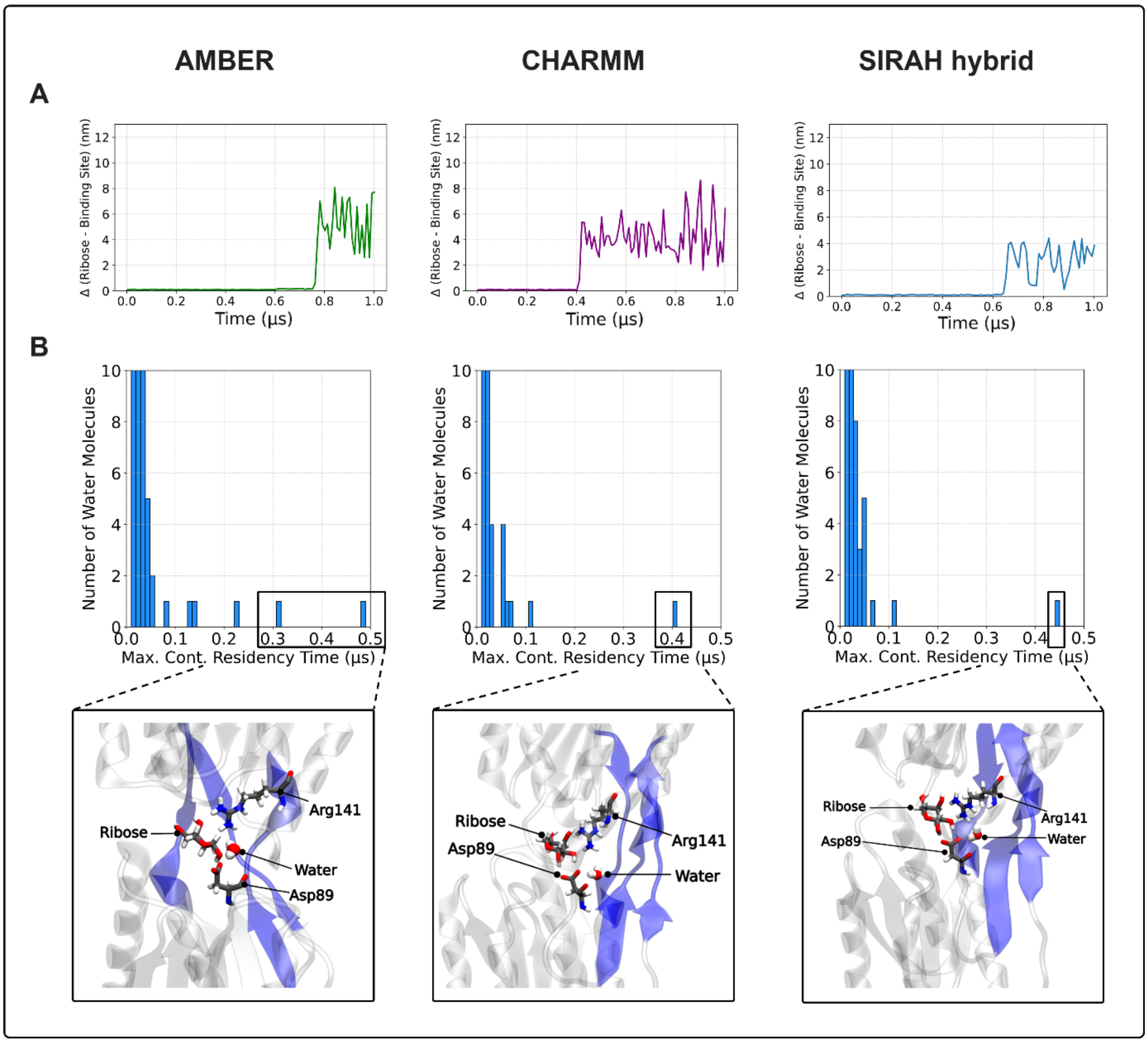


***Figure S7:*** *Dissociation of ribose from its binding pocket and the dynamics of water molecules within this region. Panel A: The distance over time between the center of mass of ribose and its initial binding site is shown over time for a single representative replica using AMBER, CHARMM, and SIRAH hybrid FFs, respectively. The increasing distance in each plot illustrates ribose leaving the binding pocket. Panel B: The histograms display the maximum continuous residency times for water molecules - until ribose dissociation - located within 0.35 nm of the initial binding site (defined as < 0.4 nm distance to ribose): while most water molecules are transient, a specific water molecule in each simulation exhibits a noticeably longer residency time (highlighted by the black boxes). In the AMBER simulation, the water molecule with the second-longest residency time subsequently occupies the position of the longest-residency water molecule. Structural snapshots further reveal that the water molecules with the longest residency time, respectively, maintain a consistent position across all FFs, forming hydrogen bonds with both the ribose and key residues of RbsB (Asp89 and Arg141).*

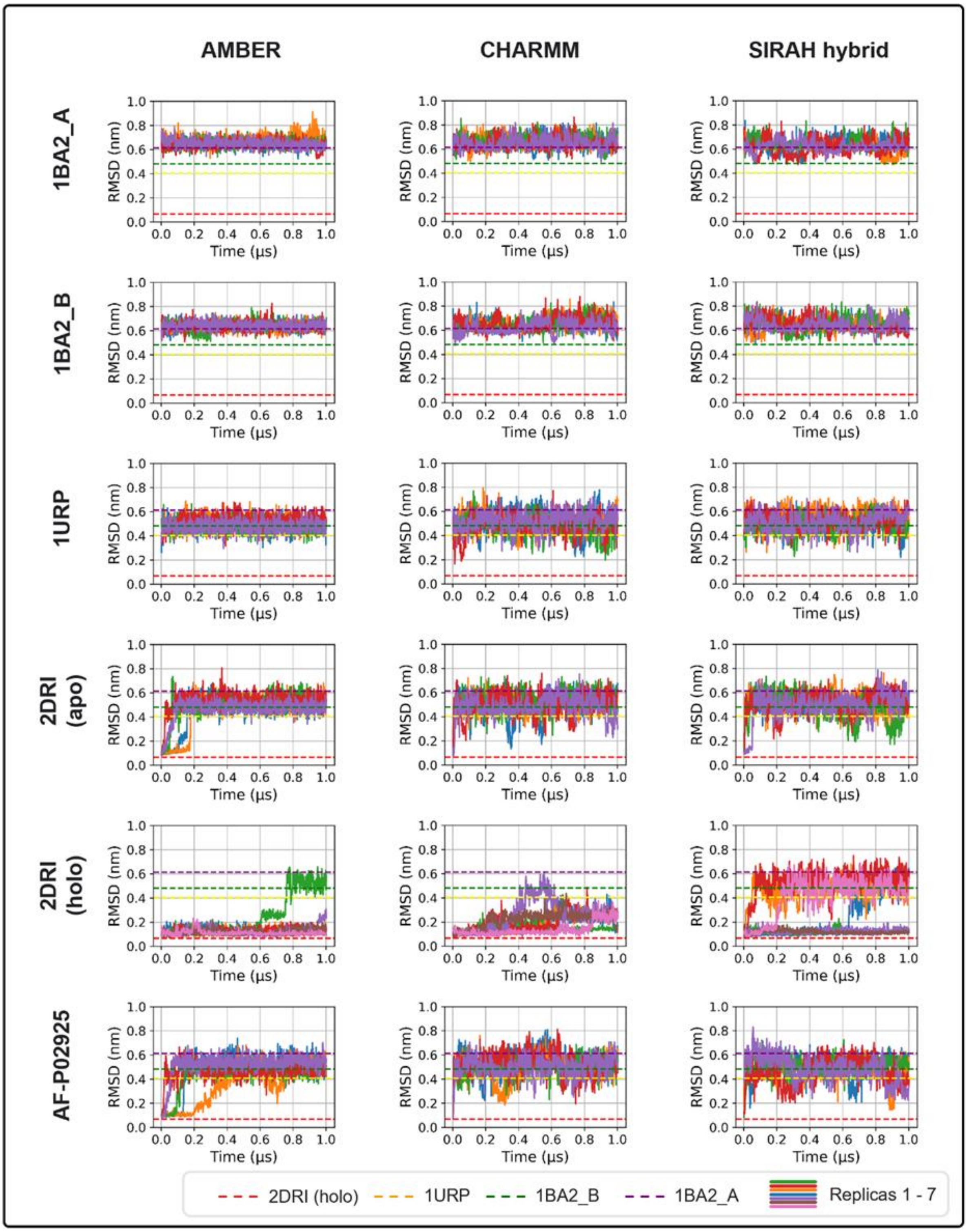


***Figure S8:*** *Root-mean-square deviation (RMSD) over time for the different open and closed conformations with the three FFs, AMBER, CHARMM and the SIRAH hybrid. The RMSD values corresponding to the X-ray structures of the protein in an open conformation are indicated by the yellow, purple and green dashed lines (pdb codes = 1URP, 1BA2; structures A & B, respectively) and closed conformation by the red dashed line (pdb code = 2DRI). The averages for 1BA2 (A and B) systems most closely aligned with the value of the X-ray structure*

*1BA2, conformation A. In contrast, the averages for 1URP and AF-P02925 systems most closely aligned with the value of the X-ray structure 1BA2, conformation B.*

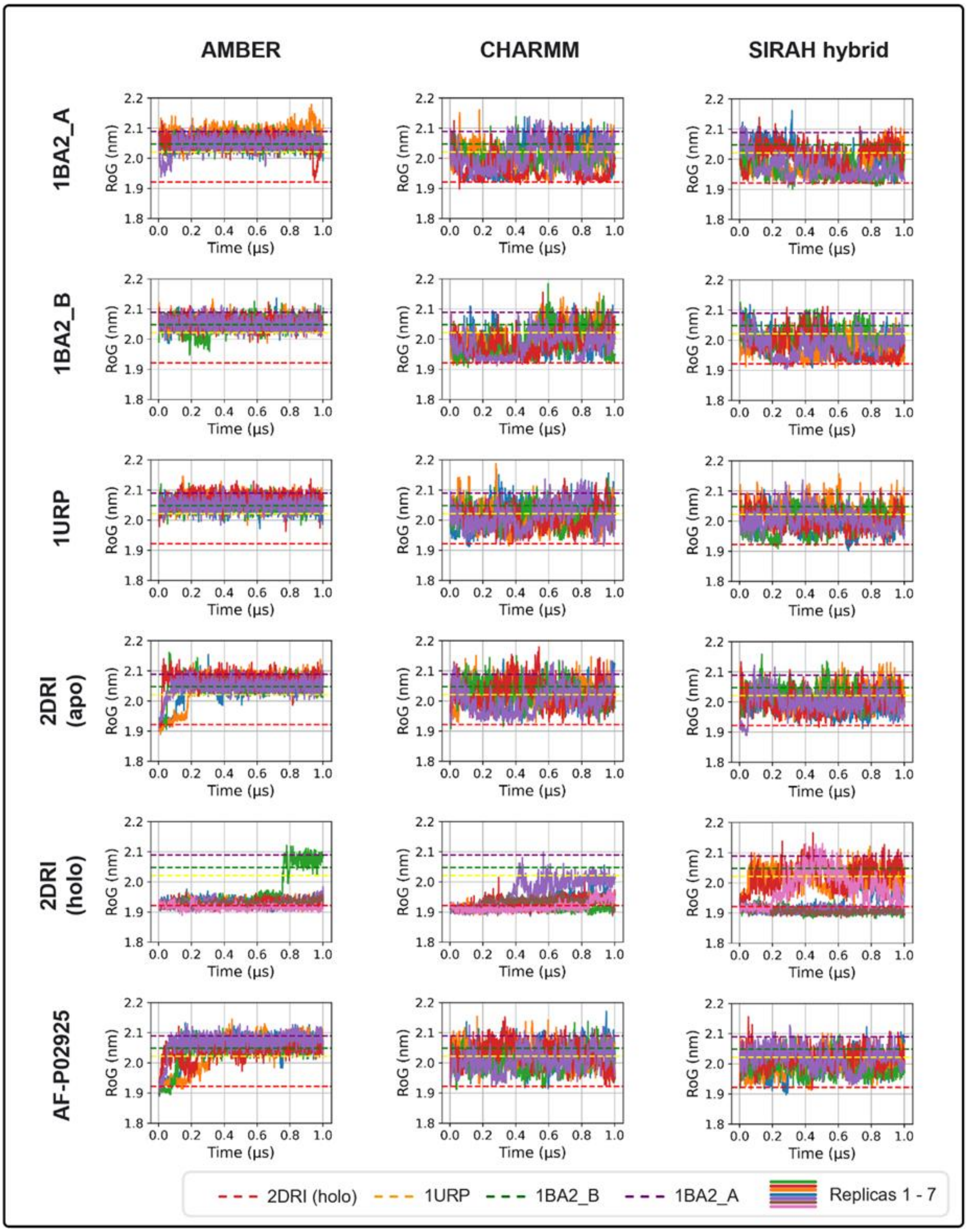


***Figure S9:*** *Radius of Gyration (RoG) over time for the different open and closed conformations with the three FFs AMBER, CHARMM, and SIRAH hybrid. The change in compactness upon opening of 2DRI and the ribose leaving can be observed across the FFs. The RoG values corresponding to the X-ray structures of the protein in an open conformation are indicated by the yellow, purple and green dashed lines (pdb codes = 1URP, 1BA2; structures A & B, respectively) and closed conformation by the red dashed line (pdb code = 2DRI).*

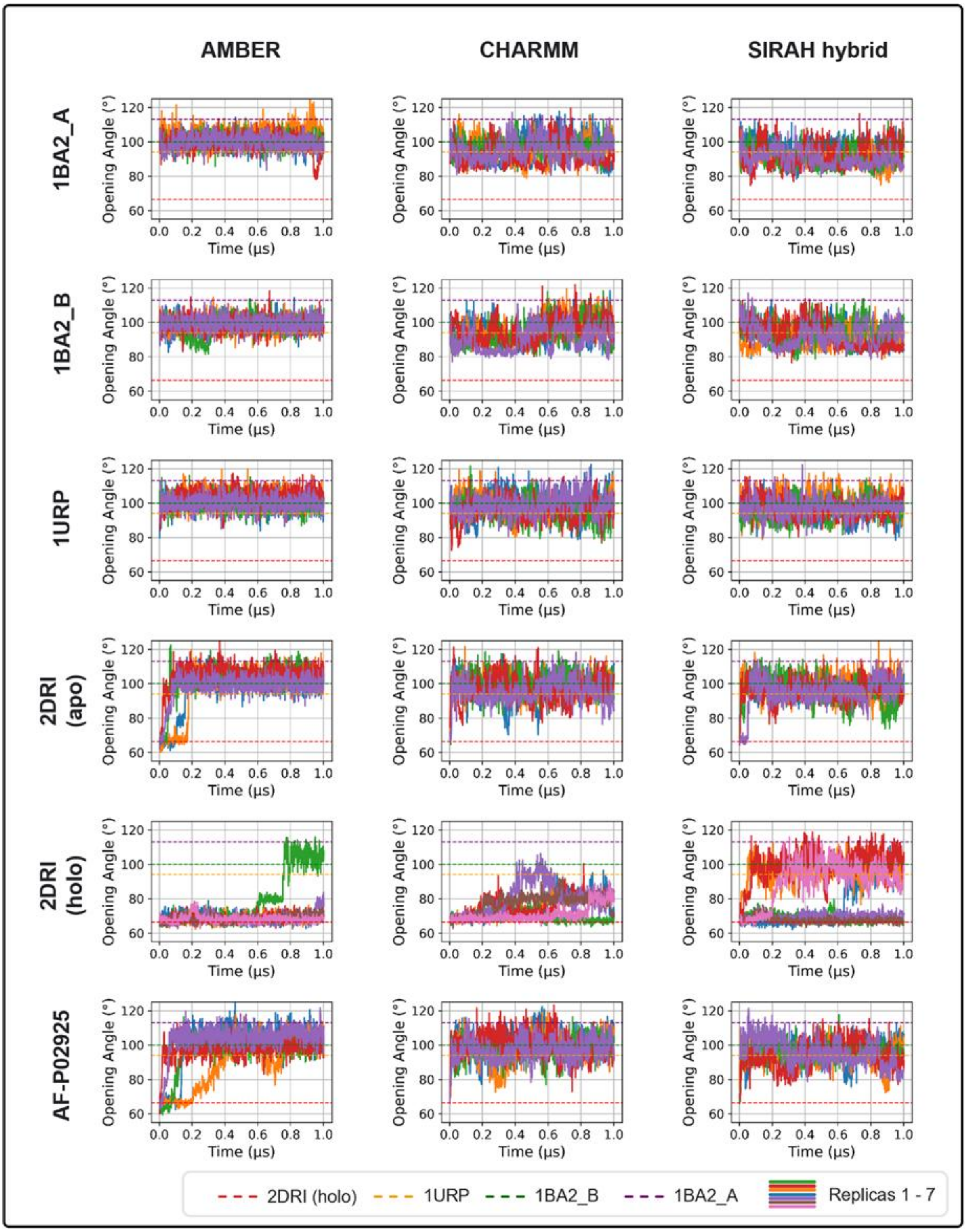


***Figure S10:*** *Opening angles over time for the different open and closed conformations with the three FFs AMBER, CHARMM, and SIRAH hybrid. The closed conformations can be seen opening at different rates. The events of ribose leaving and the opening of RbsB can be observed for 2DRI with ribose for all three FFs. Among the seven replicas per FF, one AMBER and one CHARMM simulation exhibited the fully open state, while four SIRAH hybrid FF simulations reached this conformation. The Opening angles values corresponding to the*

*X-ray structures of the protein in an open conformation are indicated by the yellow, purple and green dashed lines (pdb codes = 1URP, 1BA2; structures A & B, respectively) and closed conformation by the red dashed line (pdb code = 2DRI).*

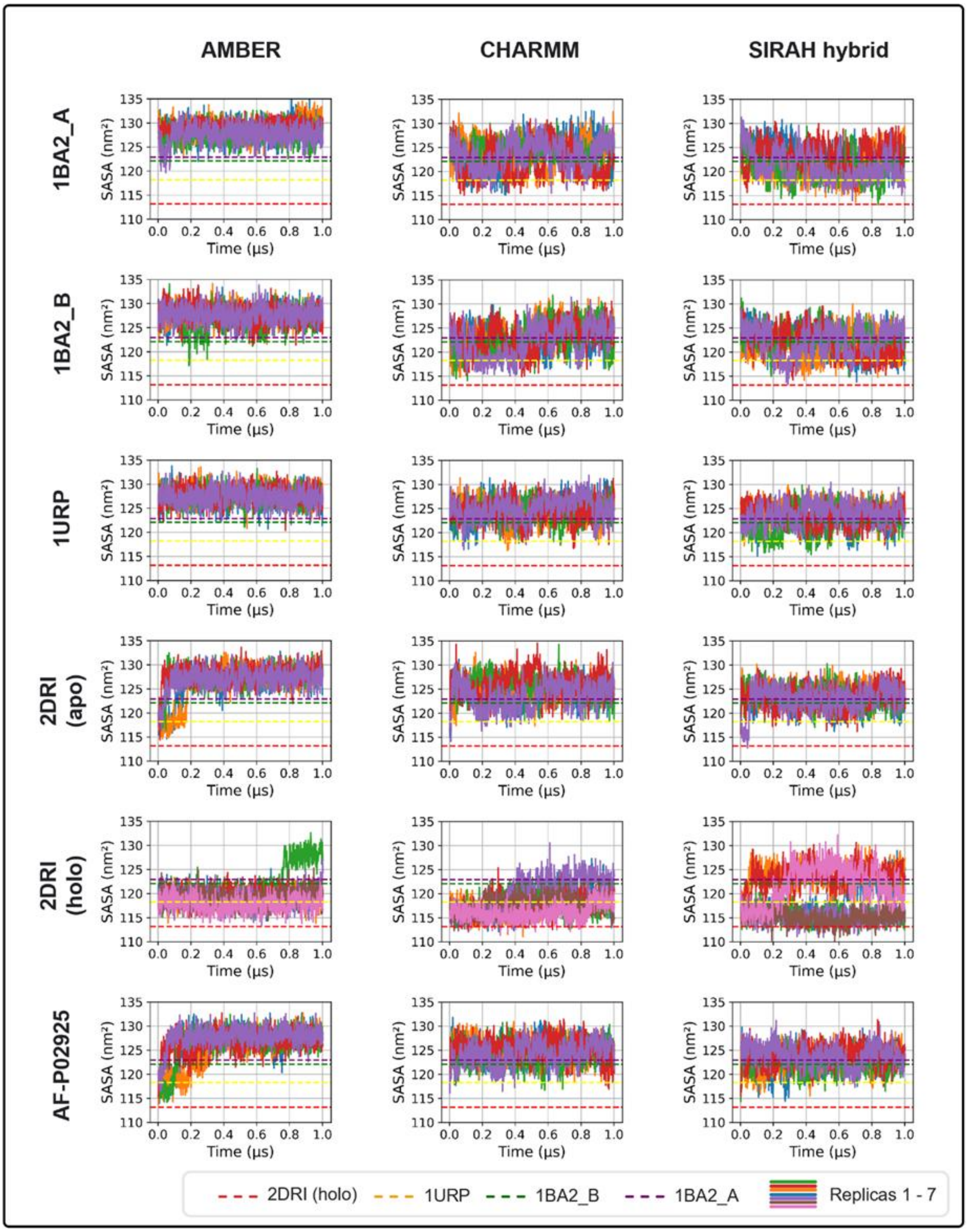


***Figure S11:*** *Solvent-accessible surface area (SASA) over time for the different open and closed conformations with the three FFs AMBER, CHARMM, and SIRAH hybrid. The SASA values corresponding to the X-ray structures of the protein in an open conformation are indicated by the yellow, purple and green dashed lines (pdb codes = 1URP, 1BA2; structures A & B, respectively) and closed conformation by the red dashed line (pdb code = 2DRI).*

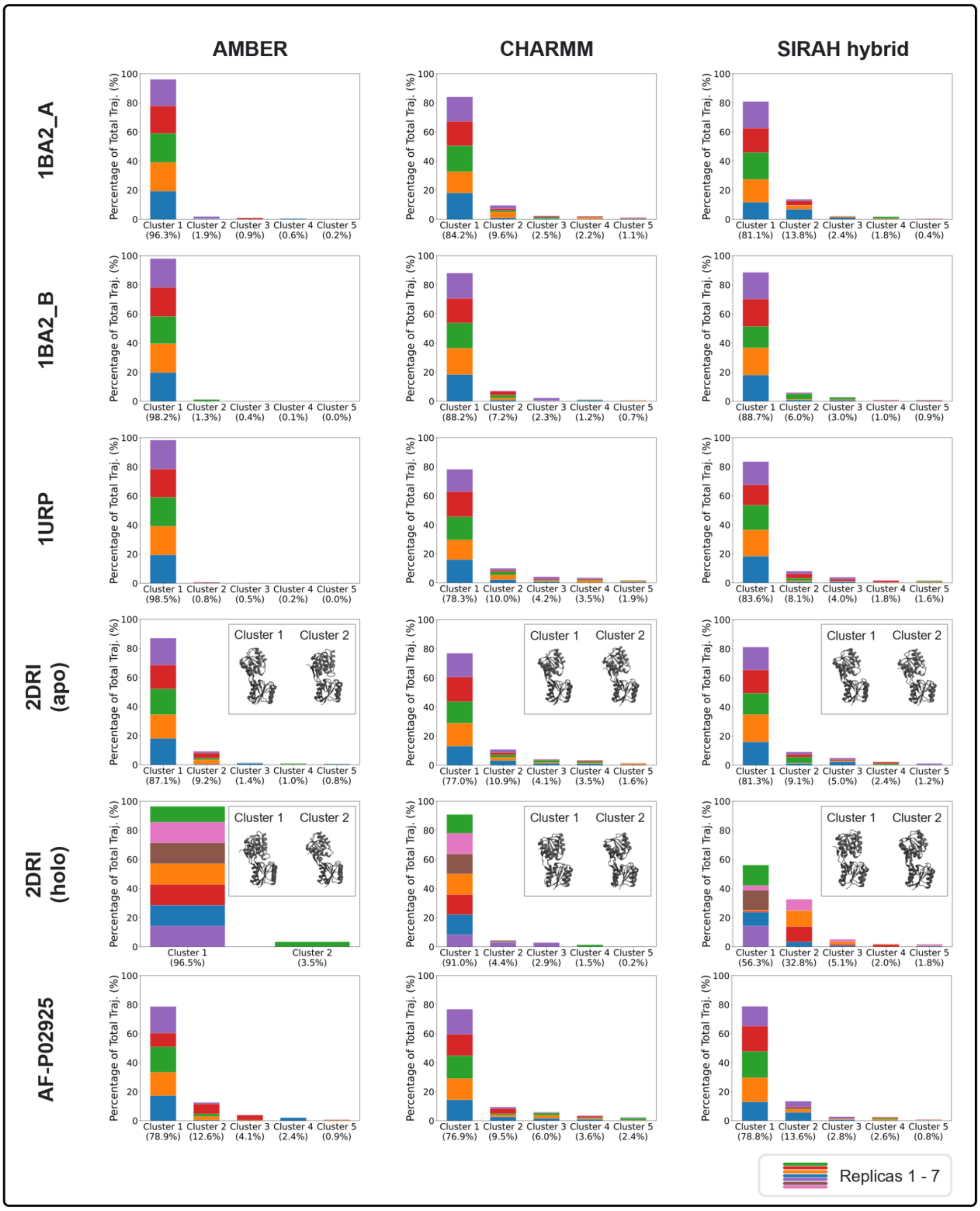


***Figure S12:*** *Relative populations of the most populated backbone root-mean-square deviation (RMSD) clusters obtained from concatenated trajectories of all independent replicas are shown for each system and FF (AMBER, CHARMM, and SIRAH hybrid). Bars report the percentage of total trajectory frames assigned to each cluster, with colors indicating the contribution from individual replicas. In all systems, the most populated clusters comprise frames originating from multiple independent replicas rather than from a single trajectory. Representative structures of the most populated clusters, corresponding to the most central*

*structures identified by the clustering algorithm, are shown as insets for selected systems (2DRI apo & holo). Less populated clusters correspond to sparsely sampled conformations characterized by extreme opening angles. The consistent inter-replica contribution to dominant clusters across FFs indicates effective exploration of shared conformational basins and supports convergence of conformational sampling over the simulated timescale.*

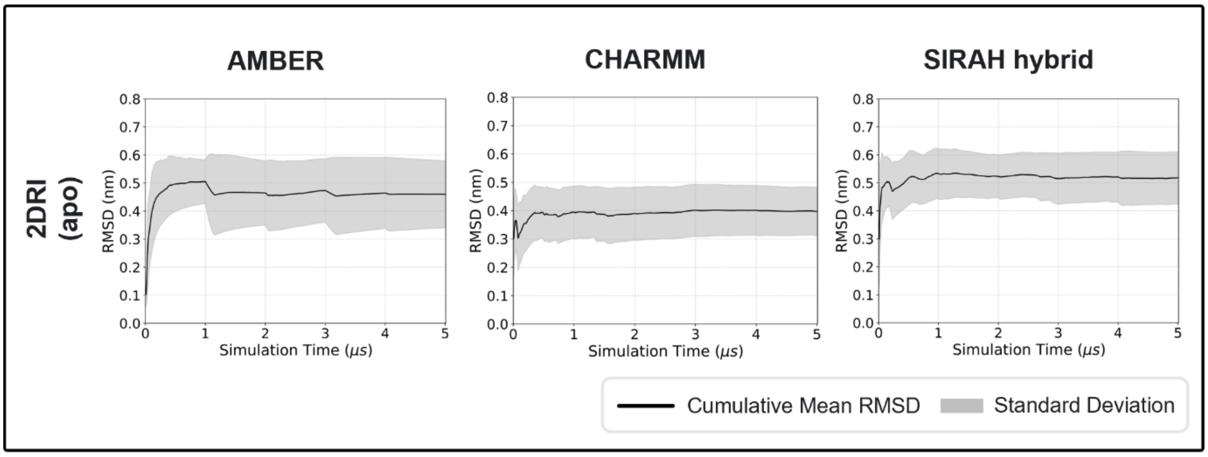


***Figure S13:*** *Cumulative mean backbone root-mean-square deviation (RMSD) and associated standard deviation as a function of simulation time for the apo 2DRI system simulated with the three FFs AMBER, CHARMM, and SIRAH hybrid. Solid lines denote the cumulative mean RMSD, while shaded regions indicate the corresponding standard deviation. The cumulative mean RMSD rapidly reached a plateau and remained stable over the remainder of the simulation time for each FF, with the standard deviation converging to a time-independent band. The absence of sustained drift and the stabilization of RMSD fluctuations indicate that the RMSD observable attains statistical stationarity over the sampled timescale.*

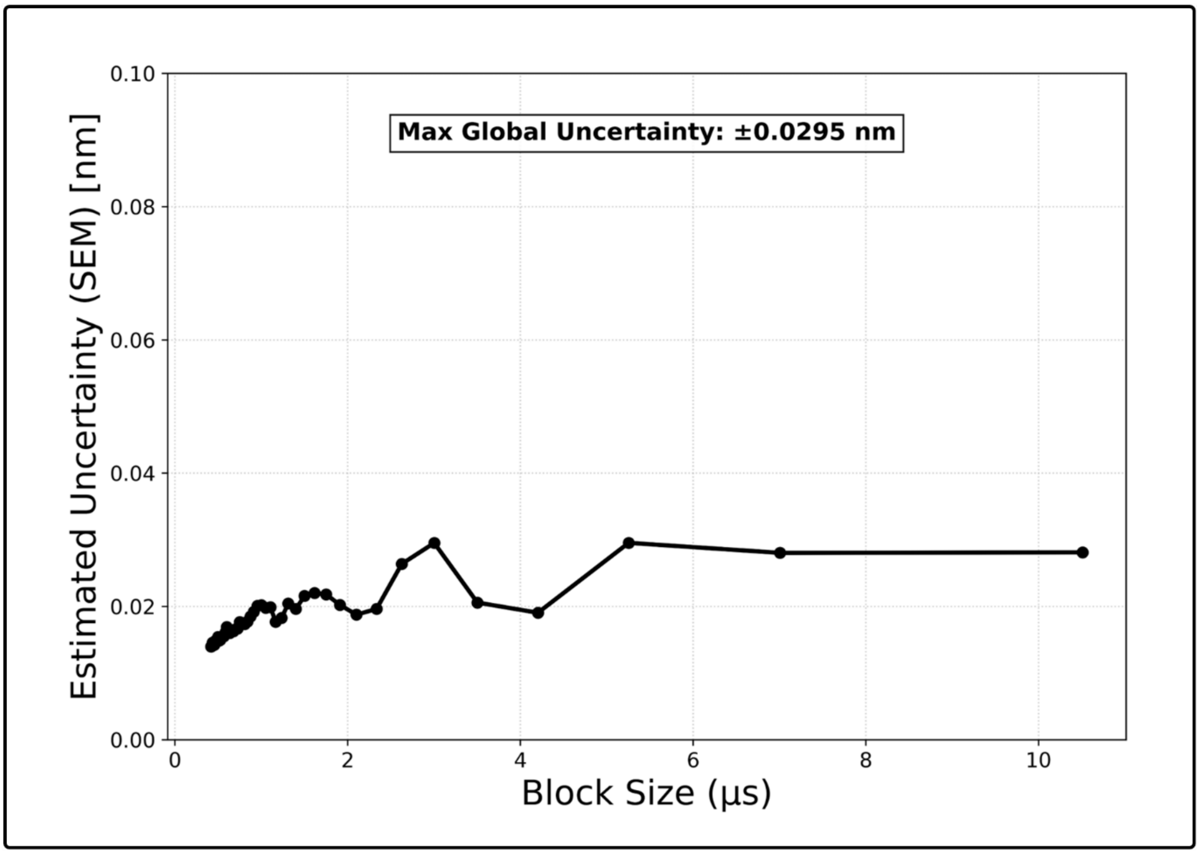


***Figure S14:*** *Time block analysis of backbone root-mean-square deviation (RMSD) for the 2DRI holo system used to estimate statistical uncertainty. RMSD data from all replicas and FFs were combined into a single 21 μs trajectory and analyzed as a function of block size. Points report the estimated uncertainty expressed as the standard error of the mean (SEM), defined as the standard deviation of block-averaged RMSD values divided by the square root of the number of blocks. The estimated uncertainty increases with block size and subsequently plateaus. Saturation of the uncertainty with increasing block size indicates that additional sampling would not substantially alter the mean, consistent with sufficient sampling of the relevant conformational states.*

## *References*

(1) Björkman, A. J.; Mowbray, S. L. Multiple Open Forms of Ribose-Binding Protein Trace the Path of Its Conformational Change. *J Mol Biol.* **1998**. https://doi.org/10.1006/jmbi.1998.1785.

(2) Björkman, A. J.; Binnie, R. A.; Zhang, H.; Cole, L. B.; Hermodson, M. A.; Mowbray, S. L. Probing Protein-Protein Interactions: The Ribose-Binding Protein in Bacterial Transport and Chemotaxis. *Journal of Biological Chemistry* **1994**, *269*, 30206–30211. https://doi.org/10.1016/s0021-9258(18)43798-2.
